# Charged amino acid variability related to N-glyco -sylation and epitopes in A/H3N2 influenza: Hem -agglutinin and neuraminidase

**DOI:** 10.1371/journal.pone.0178231

**Published:** 2017-07-14

**Authors:** Zhong-Zhou Huang, Liang Yu, Ping Huang, Li-Jun Liang, Qing Guo

**Affiliations:** 1 Department of Dermatology, Sun Yat-Sen Memorial Hospital, Sun Yat-Sen University, Guangzhou, China; 2 Key Laboratory for Emergency Pathogen Detection, Guangdong Provincial Center for Disease Control and Prevention, Guangzhou, China; 3 Department of Epidemiology, School of Public Health, Sun Yat-sen University, Guangzhou, China; University of Georgia, UNITED STATES

## Abstract

**Background:**

The A/H3N2 influenza viruses circulated in humans have been shown to undergo antigenic drift, a process in which amino acid mutations result from nucleotide substitutions. There are few reports regarding the charged amino acid mutations. The purpose of this paper is to explore the relations between charged amino acids, N-glycosylation and epitopes in hemagglutinin (HA) and neuraminidase (NA).

**Methods:**

A total of 700 HA genes (691 NA genes) of A/H3N2 viruses were chronologically analyzed for the mutational variants in amino acid features, N-glycosylation sites and epitopes since its emergence in 1968.

**Results:**

It was found that both the number of HA N-glycosylation sites and the electric charge of HA increased gradually up to 2016. The charges of HA and HA_1_ increased respectively 1.54-fold (+7.0 /+17.8) and 1.08-fold (+8.0/+16.6) and the number of NGS in nearly doubled (7/12). As great diversities occurred in 1990s, involving Epitope A, B and D mutations, the charged amino acids in Epitopes A, B, C and D in HA_1_ mutated at a high frequency in global circulating strains last decade. The charged amino acid mutations in Epitopes A (T_135_K) has shown high mutability in strains near years, resulting in a decrease of NGT_135-135_. Both K_158_N and K_160_T not only involved mutations charged in epitope B, but also caused a gain of NYT_158-160_. Epitope B and its adjacent N-glycosylation site NYT_158-160_ mutated more frequently, which might be under greater immune pressure than the rest.

**Conclusions:**

The charged amino acid mutations in A/H3N2 Influenza play a significant role in virus evolution, which might cause an important public health issue. Variability related to both the epitopes (A and B) and N-glycosylation is beneficial for understanding the evolutionary mechanisms, disease pathogenesis and vaccine research.

## Introduction

Influenza is an acute respiratory infectious disease caused by influenza virus, which affects millions of people annually and results in moderate mortality. Among the different types and subtypes of influenza virus, the A/H3N2 subtype has dominated a lot of human influenza outbreaks worldwide since its emergence in 1968 [1]. Based on influenza surveillance, H3N2 virus evolved genetically and became the dominant strain in 2014/15 season in Japan (99%) and in Europe (83%) [2,3], but accounted for only 23.9% (625/2616) in 2015/16 season in the United States [4]. Both events of A/ H3N2 epidemic occurred in 2010 (81.9%, 127/155) and 2012 influenza seasons (Feb. to Jul.) in the Southern China [5,6].

Influenza viruses are subtyped by surface glycoproteins, which include hemagglutinin (HA) and neuraminidase (NA). Viral HA performs attachment of the influenza virus to sialic acid moieties on the host cell and functions as a major antigen initiating produce host specific antibody. The HA monomer could be divided into HA_1_ and HA_2_ while former is an important functional region. Viral NA is responsible for cleaving the terminal sialic acid residues, which helps to release viruses from the host cell [7]. The function of NA is to cleave the terminal sialic acid residues present on cell surfaces and progeny virions, facilitating release of the virus from infected cells and thus playing an important role in release and spread of progeny virions. Because of the lack of proofreading activity of its polymerase, influenza virus genes mutate very frequently without genetic correction, resulting in 1–2% annual divergence of influenza strains [1,8]. The amino acid (AA) substitutions of HA_AA158_N/K/D/S/R_ and _AA160_T/K/I_ occurred in Europe 2014/15 season and those of three HA sites (A_214_S, V_239_I and N_328_S) and two NA sites (L_81_P and D_93_G) did during the 2012 season in the Southern China [2,6].

Electric charge is an important biochemical feature of HA protein, relating to HA’s antigenicity and receptor binding affinity. Mutation N_145_K in HA protein resulted in changing both antigenicity (epitope A) and receptor binding avidity, which was contributed by amino acid charge alteration [9]. Influenza M1 molecule charge drove conformational changes, leading to alterations in their electrostatic interactions [10]. Moreover, in the process of viral adsorption, a higher positive charge could promote the affinity of receptor binding domain (RBD) in HA binding to its host cell sialic acid receptors, which is highly negatively charged[11]. Evolution of the HA and NA genes has a most critical influence on influenza virus transmission, including antigenic drift and accumulation of N-glycosylation sites (NGSs) [12]. The N-glycosylation of the HA and NA acts to mask antigenic epitopes, constrain binding to host antibodies, protect the enzymatic sites of NA, and balance the activities of HA and NA [13]. Moreover, the NGSs in variants might play an even more important role in influenza virus evolution. HA epitopes (B-cell epitope) of A/H3N2 have been studied to detect mutations during each influenza season, including epitopes A, B, C, D, E, L and R [7,14]. Amino acid mutations resulted in changing in the epitope charge and interaction as the a combination of epitope sequences, pH optimization, and the additive L-arginine presented hydrophobic epitopes and their subsequent assembly into virus-like particles [15].

Herein, we sequenced the HA and NA genes of H3N2 viruses for years and analyzed the genetic and amino acid mutation and evolution including AA features, NGSs and epitopes. Our aim is to learn correlation between AA features (including charges) and NGSs/epitopes.

## Materials and methods

### Virus and gene

Human H3N2 strains isolated in Guangdong, Southern China, from 2012 to 2016 were selected based on space-time sampling [1], including 7 isolates collected in 2013, 5 in 2014, 14 in 2015 and 2 in 2016, representing 28 total isolates (GenBank Nos.: MF113163—MF113204, MF150433—MF150446). The other global 672 HA and 663 NA gene sequences were retrieved from the Influenza Virus Resource (http://www.ncbi.nlm.nih.gov/genomes/FLU) and GISAID database (http://platform.gisaid.org) used as references. A total of 700 HA genes and 691 NA genes were analyzed. The viruses used as gene references (including HA/NA) were isolated from Asia (217/218), North America (282/275), Europe (139/131) and Oceania and other (62/67) from 1968 to 2016, including A/Hong Kong/1/ 1968 (HK68) and variants, and nine vaccine strains (A/Wellington/1/2004, A/California/7/2004, A/Wisconsin/67/ 2005, A/ Brisbane/10/ 2007, A/Perth/16/2009, A/Victoria/361/2011, A/Texas/50/ 2012, A/Switzerland/ 9715293/2013 and A/Hong Kong/ 4801/2014) [16,17].

### Primer design and gene sequencing

Used Primer Premier 5.0 (Premier Biosoft International, Palo Alto, CA, USA), a set of primers was designed based on the sequences of the HA and NA genes of human H3N2 isolated during the period 2005–2011 (Table 1)[18]; the oligo DNAs were synthesized by Life Technologies (Shanghai Branch, China).

**Table 1:** A set of primers of HA and NA genes of H3N2 viruses.

Gene	HA		NA	
	Name	Sequence (5`→3`)	Name	Sequence (5`→3`)
	HF1	GCAAAAGCAGGGGATAATTC	NF1	AGCRAAAGCAGG
	HR1	TTGTTTGGCATRGTCACGTTC	NR1	TYCCATCAGTCATTACYACTG
	HF2	TATGCCTCCCTTAGGTCACTAG	NF2	AAAATGCAACTGCTAGCTTCATT
	HR2	TCATTGGRAATGCTTCCATTTGG	NR2	TTTTCTAAAATTGCGAAAGCTTAT
	HF3	AGCACMGGGAAYCTAATTGCTC	NF3	TGAYGTGTGGATGGGAAGAAC
	HR3	TGTCYTCAACATATTTCTCGAGG	NR3	AGTAGAAACAAGGAGTTTTT
	HF4	TTCAGGACCTCGAGAAATAYG		
	HR4	AGTAGAAACAAGGGTGTTTT		

#.R: A+G; M: A+C; Y: C+T.

Viral RNA molecules from H3N2 strains from Guangdong collected during 2012–2016 were extracted using a QIAamp Viral RNA mini kit (Qiagen, Venlo, The Netherlands). Reverse transcription-polymerase chain reaction (RT-PCR) assays were conducted using Qiagen Sensiscript Reverse Transcriptase and Takara PyroBest Taq. After purification using a Qiagen Gel Extraction Kit, amplicons were sequenced using an ABI PRISM 3100 Genetic Analyzer. Amplifications were performed on a 96-Well Thermal Cycler PCR system (Applied Biosystems, USA) under the following conditions: 30 min at 55°C; 2 min at 94°C; 40 cycles of 15 s at 94°C, 30 s at 55°C, and 60 s at 68°C; and a final extension of 5 min at 72°C.

### Phylogenetic and evolution analysis

Viral nucleotide sequences were analyzed by means of the Maximum Composite Likelihood and phylogenic trees were generated based on the Neighbor-Joining (NJ) in the MEGA 7.0.21 package [19]. The reliability of phylogenic trees was estimated using 1000 bootstrap replicates.

Evolutionary selection pressure was conducted using the Datamonkey web-server (www.datamonkey.org). Four different methods include the Single-likelihood ancestor counting (SLAC), Fixed effects likelihood (FEL), Internal Fixed effects likelihood (IFEL) and Random effects likelihood (REL), The SLAC, FEL, and REL are used to detect sites under selection at external branches of the phylogenetic tree, while the IFEL investigates sites along the internal branches. The SLAC is intensive for large alignments but appears to underrate the substitution rate and the REL is more suited to intermediate-sized datasets than SLAC, but it may be unsuitable for small alignments. The FEL method gives a lower rate than REL of false positives for data sets of few sequences [20]. The SLAC, FEL and IFEL were employed with the significance of 0.1.

### Amino acid (AA) charge and molecular model

The amino acids based on their charges were classified into three groups including acidic amino acids [Aspartic acid (D) and Glutamic acid (E)], basic amino acids [Lysine (K), Arginine (R) and Histidine (H)] and neutral AA. At a specific physiological pH, each acidic/basic AA is ionized alternatively with a single negative/positive net charge, respectively [11]. For HA or NA monomer and subunit, the net charge (NC) was calculated by subtracting the number of acidic AA from that of basic AA.

The molecular models of H3 HA and N2 NA was performed with the HA of A/Hong Kong/1/ 1968 (HA PDB: 4WE4 and NA PDB: 2hty) (http://zhanglab.ccmb.med.umich.edu/I-TASSER/) [21]. The three-dimensional structure was decorated with the UCSF Chimera package v1.11.2 (www.cgl.ucsf.edu/chimera/) [22].

### N-glycosylation sites (NGSs) and antigenic epitope analysis

The potential NGSs (Asn-X-Ser/Thr, X is any AA except Pro) were analyzed using the NetNGlyc 1.0 Server (http://www.cbs.dtu.dk/services/Net-NGlyc/). The scores of two pluses (++) or three pluses (+++) in the N-Glyc results were classified as strong potential NGSs (having strong potentiality for N-glycosylation). Based on the server-based analysis, ~76% of positively scored loci are modified by N-Glycans that contain Thr[21] [23].

The H3 HA protein includes five epitopes (A, B, C, D and E) and two additional conserved neutralizing epitopes (L and R) [7,14]. The amino acid substitutions may be divided into the charge+, charge- and charge ± substitutions, according to whether they increase, decrease or maintain the positive charge, respectively. During calculating epitope charge, as the number of 1968–1987 genes downloaded from GenBank were under the statistical requirements, these genes were calculated in six groups, 1968-1971(n = 20), 1972-1973(n = 19), 1974-1975(n = 18), 1976–1979 (n = 24), 1980–1983 (n = 25) and 1984–1987(n = 22). Genetic data collected after 1987 analyzed annually. To avoid sampling bias, the average epitope charge values were unweighted by the data numbers.

### Calculation and statistical analysis

Correlation and regression were statistically performed and statistical significance was depended on its significance probability (*P*<0.05), used SPSS 20.0 (SPSS Inc., Chicago, IL.). Calculated electric charge, the net charge *S*_*nc*_ = Σ^*n*^_*i*_, where *n* is the respective number of acidic AAs (D and E) and basic AAs (K, R and H) in each monomer or epitope, and *i* is each charge of the five charged AA.

## Results

### Genetic evolution and selection

**Fig 1:**
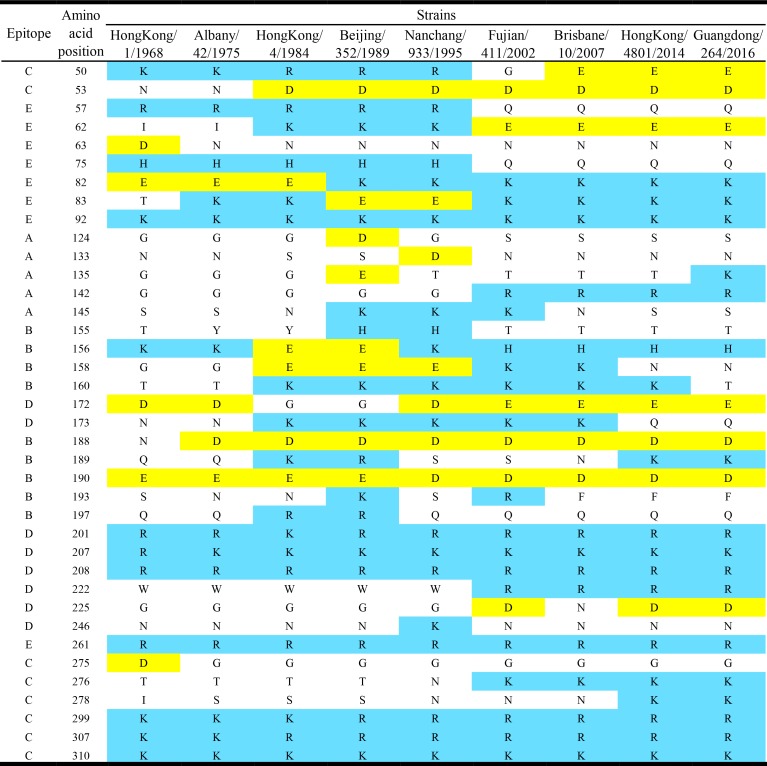
Charged amino acid mutations on HA epitopes in variants/vaccine strains. Nine variants/vaccine strains were analyzed and only charged amino acids included. Both acidic amino acids [Aspartic acid (D) and Glutamic acid (E)] and basic amino acids [Lysine (K), Arginine (R) and Histidine (H)] labeled in different colors. The epitopes identified referred to Reference 7, 14 and the article (Lees WD, et al. J Virol. 2011. doi: 10.1128/JVI.00579-11).

**Fig 2:**
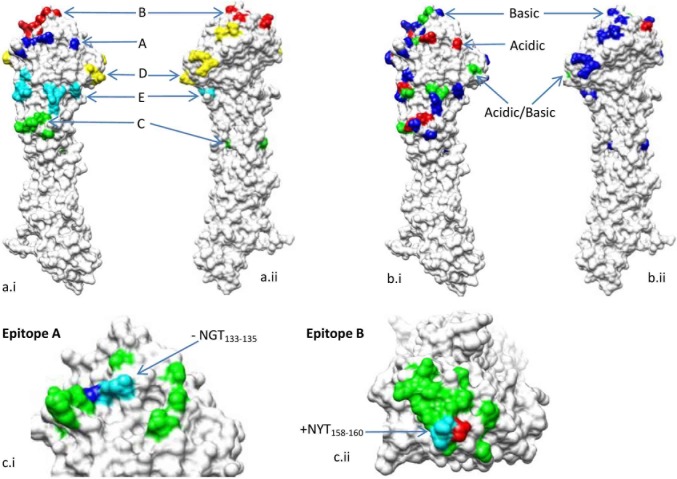
Spatial changing of charged amino acids on HA epitopes in variants/vaccine strains. a. Charged amino acids of five epitopes on HA included, Epitope A labeled in blue (124, 133, 135, 142 and 145), B in red (155, 156, 158, 160, 189, 190, 193 and 197), C in green (50, 53, 275, 276, 278, 279, 307 and 310), D in yellow (172, 173, 201, 207, 208, 222, 225 and 246) and E in cyan (57, 62, 63, 75, 82, 83, 92 and 261)(a.i and a-ii). b. The charged amino acids on epitopes of variants/vaccine strains were labeled, acidic ones in red, basic ones in blue and both acid and basic ones in green. c. T_135_K on Epitope A resulted in a decrease of NGT_133-135_, labeled in blue (T_135_K) and cyan and K_160_T on Epitope B did in an increase of NYT_158-160_, labeled in red (K_160_T) and cyan. Other amino acids on epitopes adjacent to NGS were labeled in green.

Both phylogenetic trees were generated from the HA and NA genes of the influenza H3N2 viruses isolated between 1968 and 2016. A remarkable diversification of HA genes occurred in 1993, in which many nucleotides and corresponding AA mutations were identified, including charged S_133_D and E_135_T mutations in Epitope A, charged E_156_K, R_189_S, K_193_S and R_197_Q mutations in Epitope B, charged G_172_D and N_246_K mutations in Epitope D (Fig 1). For the NA graph, similar to HA genes, the NA genes evolved chronologically and a major diversification of NA genes occurred around 1990. Last decade, the charged amino acids mutations related to epitopes included Epitope A (T_135_K), Epitope B (K_158_N, K_160_T and N_189_K), Epitope C (N_278_K) and Epitope D (K_173_Q and N_225_D). Based the variants/vaccine strains, the spatial positions of charged amino acids on HA epitopes were shown (Fig 2), key frequently mutating including G_135_E/T/K, G_158_E/K/N, T_160_K/T, N_173_K/Q, Q_189_K/R/S/N/K, G_225_D/N/D and I_278_S/ N/K.

Used the Datamonkey web-sever, the numbers of 10, 15 and 18 residues in HA genes were identified as the positive selection sites by the methods of SLAC, FEL and IFEL (*p<*0.1), respectively. Only sites 121, 137, 138, 145, 159 and 262 in HA genes were considered as the positive selection with above methods. For NA genes, only sites 370 and 435 were done as the positive selection by three methods.

### Biological charge of HA and NA

**Fig 3:**
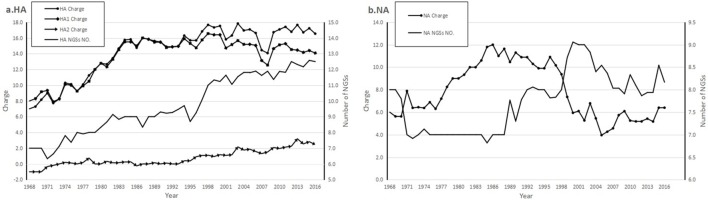
Charges and numbers of NGSs in HA and NA monomer. a. HA, b. NA. Horizontal axis marked the year and both vertical axes marked the charge values (left) and the NGS number (right). The Potential NGSs of HA and NA were analyzed using NetNGlyc 1.0 Server (http://www.cbs.dtu.dk/services/Net-NGlyc/). N-glycosylated sites predicted with “++” or “+++” score would include.

Both HA and NA charges depending on each AA charged were analyzed, whose strains isolated from 1968 to 2016 (Fig 3), in addition, that the HA could be divided into HA_1_ and HA_2_. The charges of HA_1_/HA were +8.0/+7.0 in 1968 and gradually and almost synchronously increased to +16.0/+16.0 in 1987. However, the charge of HA_1_ peaked at +16.6 in 1998, whereas HA reached the maximum +17.8 in 2003. HA_2_ net charge value was under 2 before 2003 then increased slowly and peaked at +3.2 in 2013; the latter suggested that the HA_2_ subunit had an effect on whole HA monomer charge after 2003. Compared with the charges of HA and HA_1_ in statistical regression, the coefficient of determination *R*^*2*^_*HA*:*HA1*_ = 0.924 and *R*^*2*^_*HA*:*HA2*_ = 0.503, which might indicate that the HA charges was depended mostly on HA_1_ charges.

The NA charges increased from +6.0 (1968) to +12.0 (1986), then kept around +10 (to 1998). But they decreased rapidly from +9.4 (1998) to +5.9 (2000), then it rose slightly.

### Variation of NGS

**Fig 4:**
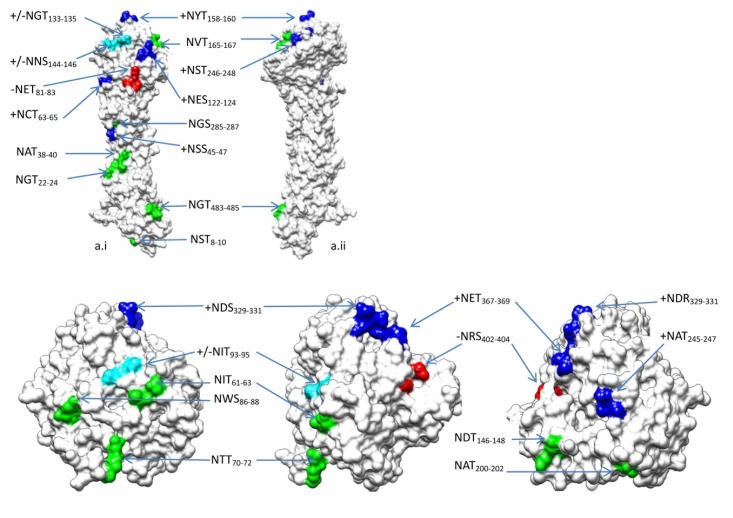
Mutations of NGSs in HA and NA of A/H3N2 on variants/vaccine strains. The mutations of NGSs on HA and NA included respectively 14 and 11 NGSs. The original NGS apearing in 1968 and preserving was labelled in green colour, then disapearing in red. The emerging NGS preserving up to date was labelled in blue colour, then disapearing in cyan.

A total of 15 potential NGSs were identified in HA genes spanning 48 years. The HA NGSs showed a gradual increase, ranging from 7 to more than 12 (14 domains). The spatial position of NGSs on HA proteins in variants/vaccine strains were shown in Fig 4. It was a special time during 1995–1998 that NGSs gained from 8.7 to 11.0. The incremental NGSs in the most strains since 1996 included NES_122-124_ and NGT_133-135_ and remained up to date. As to the HA gene of the vaccine strain A/Hong Kong/4801 /2014, a total of 11 NGSs were identified, such as NST_008-010_, et al (Table 2). It was worth noting that NGT_133-135_ became a conserved NGS since its first appearance in 1996 (95.6%, 452/473), but its uncertain potentiality accounting for 22.0% (99/452) got a lower N-Glyc score (<++), especially during 2003–2007. Remarkably, due to the mutation K_160_T, the NYT_158-160_ emerged in 2014 and got prevalent quickly worldwide (20.8% in 2014, 65.0% in 2015 and 54.2% in 2016). The NST_008-010_, NGT_022-024_, NVT_165-167_, NCT_063–065_ and NGS_285-287_ in HA genes were relatively conversed and remained stable potentiality during the whole period (Table 3), whereas the NGT_133-135_ was almost conversed and stable in previous two decades, but deleted in recent two years, including in the HA of A/ Guangdong/264 /2016. The NET_081–083_ and NCS_276-278_ occurred respectively during 1968–1975 and 1992–1997. In addition, the NGT_483-485_ was the only one identified in HA_2_, whose expression and composition in strains were nearly constant. It was correlated in statistics between the NGSs number and charge values in HA/HA1 (*r*_*NGS*:*HA charge*_ = 0.834, *p*<0.001; *r*_*NGS*:*HA1 charge*_ = 0.677, *p*<0.001), which indicated that the amino acid mutations involved charge changing were highly correlated with NGS domains here.

**Table 2:** Potential N-glycosylation of HA and NA genes of H3N2 viruses^a^.

HA				NA			
Position	Amino acid sequence	Proportion (%)	N-Glyc result^b^	Position	Amino acid sequence	Proportion (%)	N-Glyc result
008–010	NST*	96.7	+++	043–045	NSS	1.3	-
022–024	NGT*	99.9	++	061–063	NIT*	98.1	+++/++
038–040	NAT*	99.9	+/-		N(V/T/M)T	1.9	+++
045–047	NSS*	22.7	+/-	069–071	NNT	2.7	+
063–065	NCT*	95.1	++	070–072	NTT*	99.1	+
081–083	NET	5.0	+++	086–088	NWS*	99.3	++/+
122–124	NES*	64.3	-	093–095	NIT	16.2	++
126–128	NWT*	83.7	+	146–148	NDT	65.4	++
133–135	NGT*	64.6	++/+		NNT*	33.1	+
144–146	NNS	24.9	-	200–202	NAT*	99.9	-
	NKS	7.4	+	234–236	NGT*	99.8	+++
	NSS	12.0	+/-	245–247	NAT	5.4	+
158–160	NYT	6.4	+	329–331	NDS*	71.9	-
165–167	NVT*	99.3	++	367–369	NET*	25.3	+
246–248	NST*	82.7	+	402–404	NRS	78.4	-
276–278	NCS	4.0	++				
285–287	NGS*	99.4	+++				
483–485	NGT*	99.9	+				

*These NGS sequences were consistent with the HA and NA genes of vaccine strain A/Hong Kong/4801/2014.

a.Potential N-glycosylation sites were analyzed by using the NetNGlyc 1.0 Server.

b.The N-glycosylation site predicted with “++” or “+++” score would be identified as a strong potential one with asparagine N-glycosylated.

The NGSs of NA genes changed more slightly than those of HA, including 8–11 NGSs (Fig 4). An acquisition of NIT_093-095_ was due to point mutation K_93_N during 1997–2000 and a loss was due to point mutation N_93_D/G since 2008. There were 8 NGSs emerging in NA of A/Hong Kong/4801/2014, such as NIT_061-063_, et al (Table 2). The NDT_146-148_ used to be a highly conserved and a potential one up to 2008 and the D_147_N mutation resulted in a reduction in the glycosylation potentiality (++→+). Both NIT_061-063_ and NGT_234-236_ were the strongest and conservative glycosylated potential since 1968 (Tables 2 and 3). A lowly negative correlation was shown statistically between the number of NA NGSs and net charge value (*r*_*NGS*:*NA charge*_ = -0.491, *p*<0.001).

**Table 3:** Variability of strong potential N-glycosylation sites for years*.

Gene	HA										NA					
Year		68–75	76–81	82–87	88–92	93–96	97–02	03–07	08–11	12–16		68–79	80–89	90–98	99–07	08–16
No.		57	36	35	59	52	83	117	106	155		92	70	107	177	245
Percentage																
	Position										Position					
	008–010	89.3	86.1	100	100	100	100	97.4	100	100	061–063	100	98.6	100	100	98.8
	022–024	100	100	100	100	100	100	100	100	99.4	086–088	0	0	1.9	18.2	1.2
	063–065	42.9	88.9	100	100	100	100	100	100	100	093–095	0	0	0	63.6	0
	081–083	48.2	0	0	0	0	0	0	0	0	146–148	100	100	98.1	84.1	0
	133–135	0	0	0	0	5.8	90.2	34.2	96.2	85.8	234–236	100	100	99	100	100
	165–167	100	100	100	100	100	98.9	98.3	100	100						
	276–278	0	0	0	3.4	42.3	1.2	0	0	0						
	285–287	100	97.2	100	100	96.2	100	100	98.1	100						

*.Only N-glycosylation sites with “++” or “+++” scores of N-Glyc results (strong potential N-glycosylation sites) included.

### Mutations of AA and charge in epitopes

Referred to the reference of HA gene of A/Hong Kong/4801/2014, mutations of HA antigenic epitopes were identified during 2007–2016 (Table 4). The prevalent mutations included epitope A (R_142_G and S_144_N), epitope B (Y_159_F/S and K_160_T), epitope C (D_053_N and K_278_N) and epitope E (Y_094_H). Most notably, mutations Q_197_K (epitope B) and N_171_K (epitope L) emerged and became prevalent in 2016. Among the 17 mutations (Table 4), 88% (15/17) of them contained charged variation (+ or -). For the strains isolated in Guangdong, the Southern China, the mutations of R_142_G (40.6%, 13/32, epitope A), S_144_N (59.4%, 19/32, A), Y_159_F (46.9%, 15/32, B) and K_160_T (21.8%, 7/32, B) occurred during 2012–2016.

**Table 4:** Prevalence mutations of H3 epitopes from 2007 to 2016^a^.

Year			2007	2008	2009	2010	2011	2012	2013	2014	2015	2016
Sample no.			29	27	33	24	22	32	35	24	40	24
Epitope	Residue	Charge^b^	Mutated sites proportions (%)									
A	T_135_K	+	0	0	0	0	0	0	0	0	0	4.2
A	R_142_G	-	13.8	0	0	0	0	9.4	54.3	62.5	12.5	58.3
A	S_144_N/K^c^	±/+	89.7/0	100/0	45.5/54.5	83.3/16.7	95.5/4.5	100/0	97.1/0	70.8/0	15.0/0	41.7/0
A	S_145_N	±	100	100	96.9	100	90.9	56.3	2.9	0	0	4.2
B	N_158_K	+	89.7	85.2	18.2	0	4.5	0	2.9	0	0	0
B	Y_159_F/S	±	93.1/3.4	100/0	100/0	100/0	100/0	100/0	94.3/0	32.4/29.2	12.5/2.5	0/41.7
B	K_160_T	-	0	0	0	0	0	0	0	20.8	75	58.3
B	K_189_N	-	96.6	100	24.2	0	0	0	0	0	0	0
B	Q_197_K	+	0	0	0	0	0	0	0	0	0	33.3
C	D_053_N	+	7	0	0	54.2	63.6	25	0	8.7	5	4.2
C	K_278_N	-	100	96.3	100	95.8	90.9	50	8.6	0	0	0
D,L	Q_173_K/E	+/-	69.0/17.2	11.1/22.2	0/0	0/0	0/0	0/0	2.9/0	0/0	0/0	0/0
E	E_062_K	+	3.4	0	54.5	16.7	4.5	0	0	8.3	7.5	0
E	Y_094_H	+	0	0	0	50	63.6	25	2.9	8.3	2.5	0
L	N_171_K	+	3.4	0	0	0	0	0	0	0	7.5	20.8

a. The HA gene of the vaccine strain A/Hong Kong/4801/2014 were used as the reference.

b. The charged mutations were shown as following, + (additional positive charge),—(additional negative charge) and ± (neutral).

c. If there were multiple common mutations in a residue, a “/” would be used to separate the mutations and their proportions respectively.

**Fig 5:**
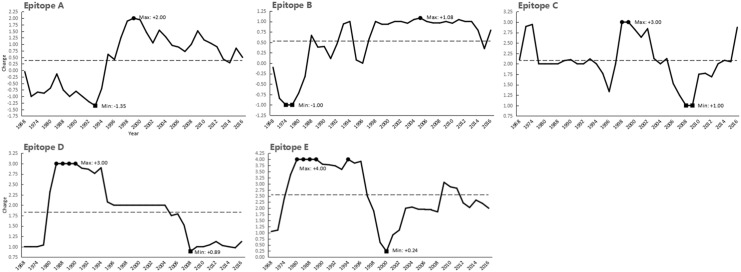
Electric charges of antigenic epitopes in HA1. The chronological changes of charge of five HA antigenic epitopes (A to E) were displayed separately. The dashed line(-), dot(●) and block(■) showed respectively the average, maximum and minimum charge values while the average charge levels in different epitopes were in diversities. As the less genes included in GenBank in the first two decades, data from 1968 to 1987 were divided into 6 groups, including 1968-1971(n = 20), 1972-1973(n = 19), 1974-1975(n = 18), 1976-1979(n = 24), 1980–1983 (n = 25) and 1984-1987(n = 22). The data during 1988–2016 were annually analyzed. The average charge of each epitope was unweighted by the number.

Electric charges of epitopes A-E have been modified greatly since 1968 up to date (Fig 5). The net charges of epitopes featured following. 1) The charge values of all five epitopes changed greatly during the period of 1993–2000, especially epitope A, C and E. 2) For epitope A, its charges reached the minimum of -1.35 in 1993 and quickly increased to its maximum (+2.00) in 1999, then went into wavelike decline; conversely, charges of epitope E dropped from its peak +4.00 (1994) then down to +0.24 (2000). 3) Charges of epitope B showed an overall increase in the period of 1974–1994 (-1.00→+1.00), being a V-shaped curve in late 1990s, then kept around at ~+1.00 until 2014. 4) The charges of epitope C underwent a sharp V-shaped curved variation in 1990s then peaked at +3.00 in 1998, while that of epitope D varied in a Z-shaped curve during 1984–2004 (1984–1994: ~+3.00, 1995: +2.08, 1996–2004: ~+2.00), where both charges of epitope C and D hit bottom in 2008 (+1.00/+0.89), then the former sharply got up while the latter slightly rose.

As to average and standard deviation (SD) values, the charges of epitope A (${\bar{\text{X}}}_{\text{A}}$ = 0.38 ± 1.02) and epitope E (${\bar{\text{X}}}_{\text{E}}$ = 2.55 ± 1.12) varied greatly, while epitope C (${\bar{\text{X}}}_{\text{C}}$ = 2.08 ± 0.53) was relatively conserved. However, great variability has been shown in epitope B and C charges in the last 3–6 years, while average charges of the former during 2013–2016 showed a sharp “V”-shape curve and the latter increased rapidly from +1.00 (2009) to +2.88 (2016). Among these epitope charges, the correlation coefficients were obtained between A and B (*r*_*A*:*B*_ = 0.603, *P*<0.001), A and E (*r*_*A*:*E*_ = -0.630, *P*<0.001), and D and E (*r*_D:E_ = 0.521, *P* = 0.001). It indicated that the positive correlation between the epitope A and B charges, the negative correlation between the epitope A and E charges to some degree, and the slight positive correlation between epitope D and E charges.

## Discussion

Influenza A/H3N2 has been circulating globally for nearly five decades and has resulted in many epidemics and deaths. According to a study in Germany, influenza- associated deaths per 100,000 persons in West Germany increased to 25.3 in 1989–1990 and 22.4 in 1995–1996, which was three- to five-fold more than usual during influenza seasons [24]. These findings corresponded to our phylogenetic diversities of HA and NA genes during the period 1990–1993, in which these genes were mutated greatly, including the charged amino acid mutations in Epitope A, B and D. Throughout for evolutionary pathway, the positive selection sites 121, 137, 138, 145, 159 and 262 in HA in this study have a remarkable impact on HA evolution [6]. Beside human H3N2 Influenza, 18 human infections linked to swine H3N2 (H3N2v) occurred in Michigan and Ohio, July–August 2016 [25]. In the Eastern China, 8.7% samples (87/1000) were identified as positive influenza and seven viruses were determined as H3 subtype AIV based on the HI results [26].

Missense mutations in nucleotide could give rise to amino acid (AA) substitutions. Charged AA (either acidic or basic one) mutations could alter the physical characteristics of a specific domain or the whole protein, furthermore some mutations resulted in a gain or loss of NGSs. Compared to strain A/Hong Kong/1/ 1968, the HA charges in this study increased and peaked at 1.54-fold and HA_1_ charges peaked at 1.08-fold, while the number of NGSs in HA were nearly doubled. This present results were similar to a previous ones, whose data only up to 2010 [12].

Electrostatic interactions are important for the viral RBD binds to the host cell sialic acid receptors. However, viral N-glycans could shield the RBD, and the highly negatively charged sialic acids and sulfuric acids on the viral N-glycans might cause electrostatic repulsion of the host cell, which has an adverse influence on the HA binding function[11]. Since the positive charge of HA1 appears to exert a beneficial effect for viral adsorption, it is possible that the increased positive charge of HA1 was to neutralize the deleterious effect from hyper-glycosylation during evolution of human A/H3N2 virus. The HA_1_ is positively charged in this study and the HA/HA1 charge values were highly correlated with NGSs number (*r*_*NGS*: *HA charge*_ = 0.834; *r*_*NGS*:*HA1 charge*_ = 0.677), which suggested that HA/HA_1_ amino acid mutations related to their charge changing mainly focused on N-glycan modification.

Some novel NGSs (such as Asn-91) might be useful candidates for functional analyses to identify innovative genetic modifications for beneficial phenotypes acquired in human lineages [27]. For H3 HA genes here, a gain of the NWT_126-128_ glycosylation site is interesting in the context of biological evolution, but the one plus (+) potential reduced its significance. The NGSs of NA were more conserved than those of HA, which suggested that NA undergo less immune pressure than HA. It was the current opinion that an increase of HA NGSs in A/H3N2 might lead to reduction of viral virulence and decline in the severity of illness [28].

A B-cell epitope is defined as a region of an antigen recognized by either a particular B-cell receptor or that can subsequently elicit antibody in a humoral response. Epitopes A, B, C, E and L in HA genes of A/H3N2 (Table 4) have mutated to great degree in this study and most antigenicity-determining AA mutations adjacent to RBD, including sites HA_145, 155, 156, 158, 159, 189 and 193, were located within epitope B except 145 (Epitope A) [29]. Amino acids in epitope B in this study were the most unstable during the last decade and it concluded following, 1) five prevalent mutations were N_158_K, Y159F/S, K160T, K189N and Q197K; 2) four of the five mutations contained charged modification; 3) mutations in position 158, 159 and 160 resulted in the gain of NGS NYT_158-160_. It’s noteworthy that both K_158_N and K_160_T were not only charged AA mutations in epitope B, but also caused a gain of NYT_158-160_. Previous study have shown that epitope B played a vital role in both antigenic phenotype and receptor specificity [30]. Mutation T_135_K occurred in epitope A, resulting in deleting a NGT_135-135_, which might be a significant mutation as well. The 1990s was also the special time for all epitopes’ net charges, which coincided with our phylogenetic diversities of HA genes. Epitopes A and B, including residues of 135, 142, 144, 159 and 160, also showed high mutability in strains isolated from Guangdong, Southern China during 2012–2016.

Antigenic mutations are based on amino acid substitutions, where the net charge value and the order in AAs for each epitope are crucial. Just like the relation between key and lock, the charged AAs located on epitope must electrically and spatially correspond to that located on antibody’s complementarity determining region, even if the general net charge is appropriate. Some opposite charged mutations emerging in one epitope simultaneously, such as K160T and Q197K (epitope B) and D_053_N and K_278_N (epitope C), might cause electrical repulsion of antibody binding, while the synthetic effect on net charge was neutral. Besides mutations in epitope B, the R_142_G (A), D_053_N (C), K_278_N (C), Y_094_H (E) and N_171_K (L) were prevalent or emerged charged mutations in recent five years as well. The accumulation of antigenic epitope mutations could, to a great extent, spark a local or provincial epidemic and/or outbreak [6,30]. Overall, amino acid mutations could initiate changes in NGSs and antigenic epitopes, which influenced viral pathogenicity towards the host.

The A/H3N2 virus circulated and dominated worldwide for more than four decades, meantime it still evolves rapidly at the genetic level. Due to its polymerase and segment reassortment, HA segments originated from equine H3 influenza might become associated with cross-species transmission even contribute to appearance of new strains [31, 32]. The HA genes genetically mutated more rapidly than the NA genes so far, especially in missense mutations, suggesting that the HA gene was under immune pressure to strive for surviving. The charged amino acids mutations including Epitope A (T_135_K), Epitope B (K_158_N and K_160_T), Epitope C (Q_311_H) and Epitope D (K_173_Q and N_225_D) during last decade. We should continue to survey that A/H3N2 variations in AA features, NGSs and antigenic epitopes of could, to some degree, be sufficient to evade immune protection in humans. Molecular monitoring of NGSs and antigenic epitopes of the influenza A/H3N2 virus is beneficial for understanding the evolutionary mechanisms that govern influenza viruses. Moreover, monitoring of viral charge might enlighten vaccine development, including the selection of vaccine delivery vector and immunologic adjuvant.

## Supporting information

S1 Fig HA and NA trees. (TIF)

S2 Fig Spatial changing of charged amino acids on HA epitopes. (TIF)

S3 Fig Mutations of NGS in HA genes. (TIF)

S4 Fig Mutations of NGS in NA genes. (TIF)

S5 Fig Accession numbers for GenBank and GISAID. (PDF)
