# Supplementary figures and images for: Charged amino acid variability related to N-glyco -sylation and epitopes in A/H3N2 influenza: Hem -agglutinin and neuraminidase
Source: PLoS One. 2017 Jul 14;12(7):e0178231. doi: 10.1371/journal.pone.0178231 (PMC5510802; doi:10.1371/journal.pone.0178231)

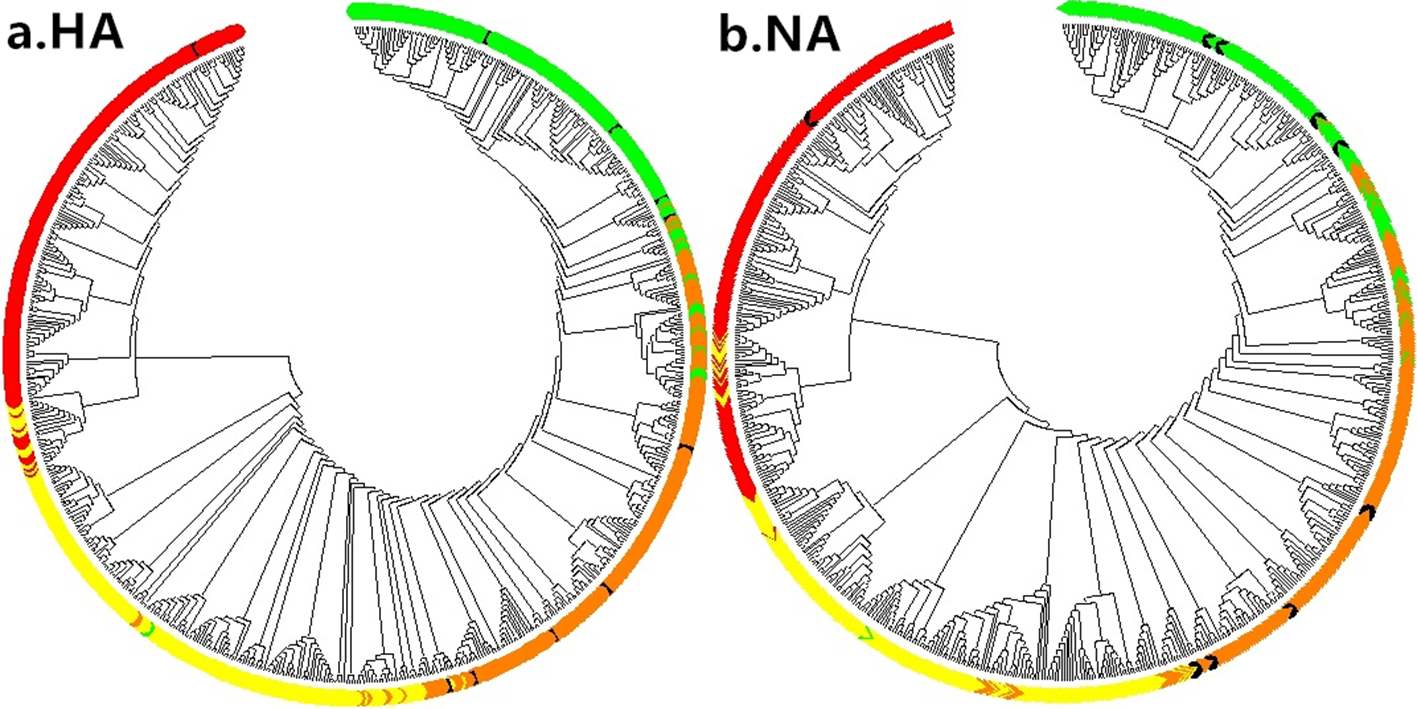

Supplement: S1 Fig — (TIF) [file pone.0178231.s001.tif]

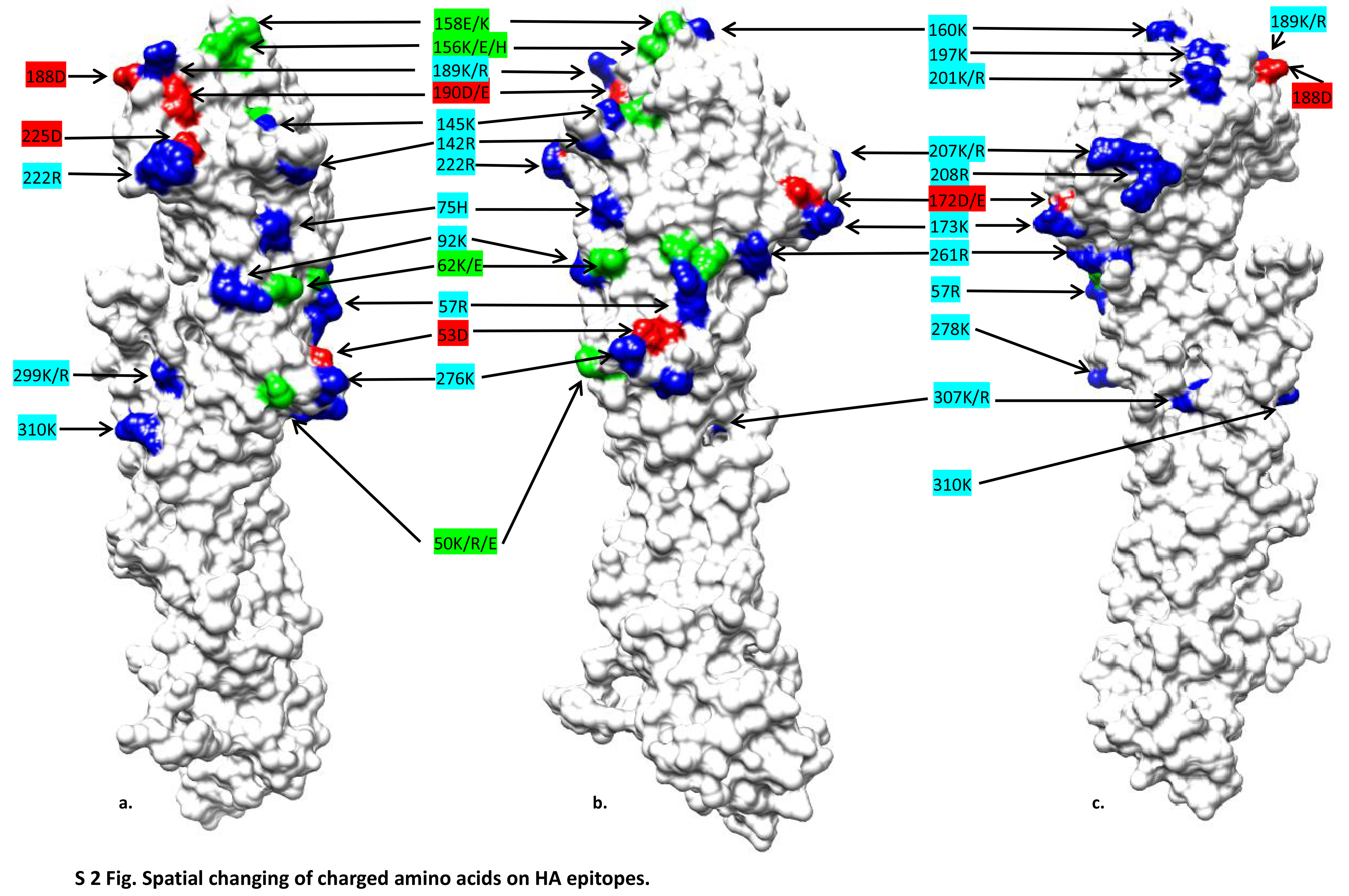

Supplement: S2 Fig — (TIF) [file pone.0178231.s002.tif]

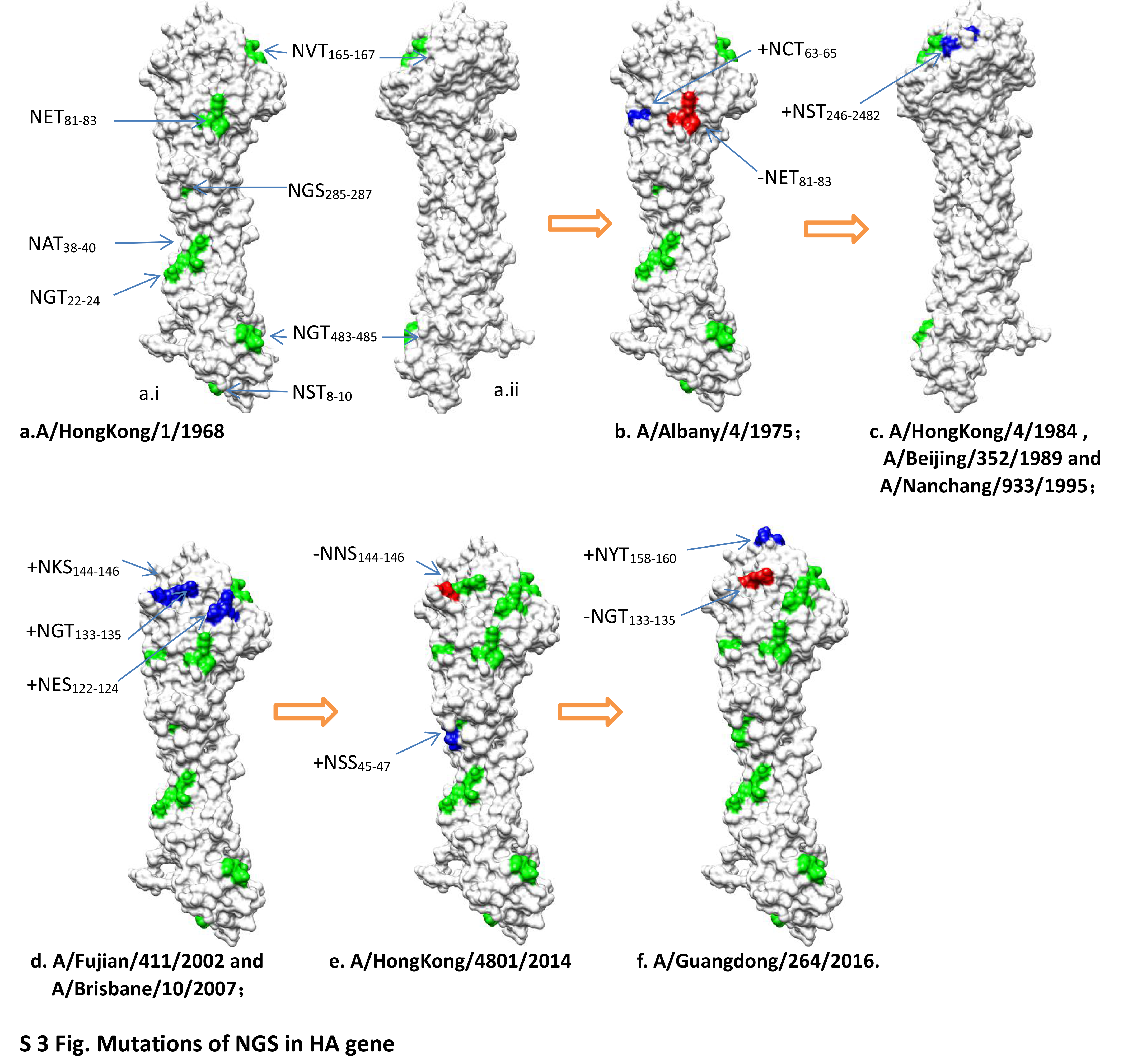

Supplement: S3 Fig — (TIF) [file pone.0178231.s003.tif]

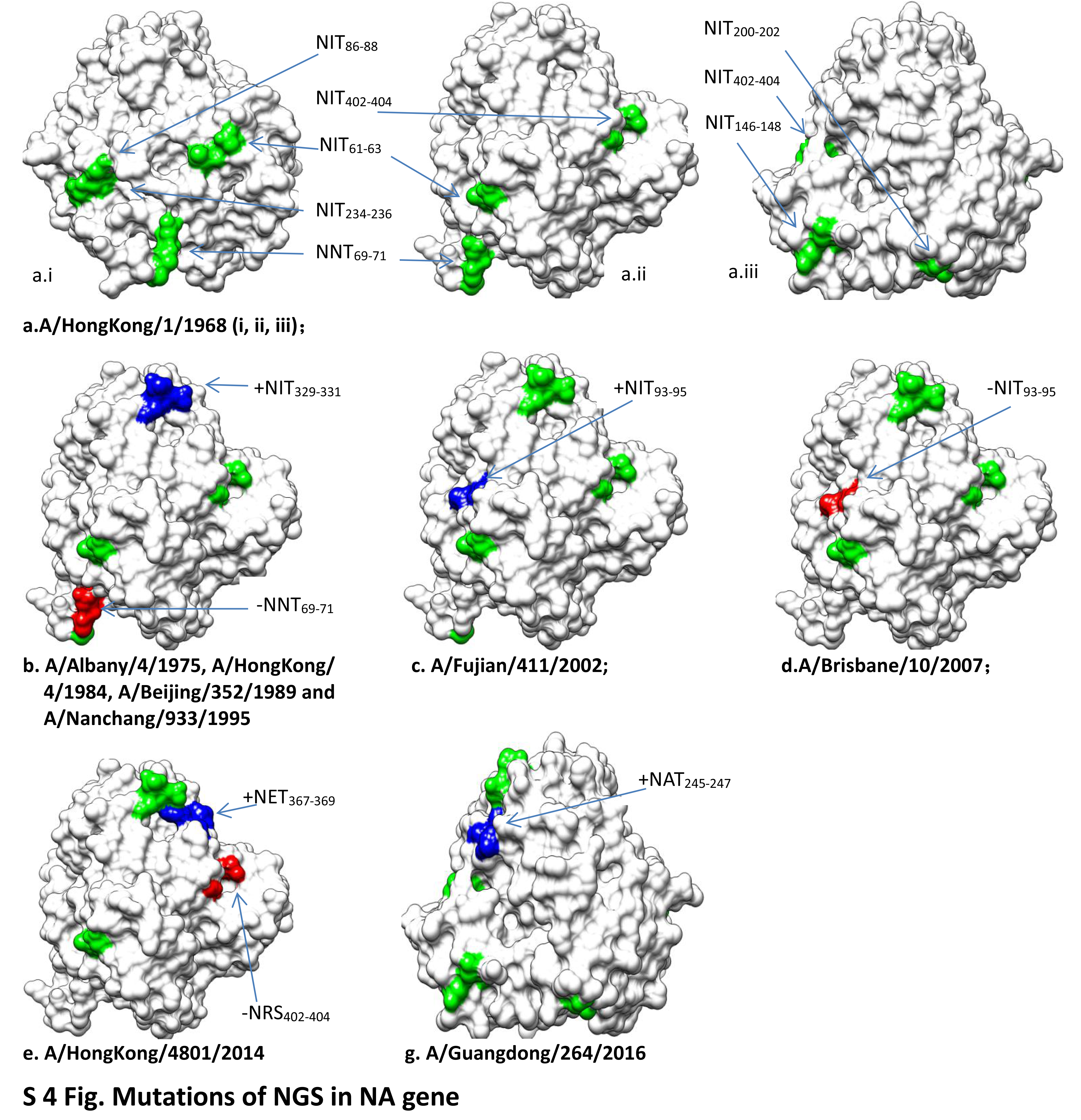

Supplement: S4 Fig — (TIF) [file pone.0178231.s004.tif]
