# Supplementary material for: Charged amino acid variability related to N-glyco -sylation and epitopes in A/H3N2 influenza: Hem -agglutinin and neuraminidase
Source: PLoS One. 2017 Jul 14;12(7):e0178231. doi: 10.1371/journal.pone.0178231 (PMC5510802; doi:10.1371/journal.pone.0178231)
Supplement: S5 Fig — (PDF) [file pone.0178231.s005.pdf]

S 5. A list of Accession Nos of HA and NA genes of H3N2 during 1968 - 2016*

HA

Year	Name	No
1968	Hong_Kong/1/1968	AF348176
1969	Albany/1/1969	CY019899
1969	Albany/3/1969	CY019915
1969	Albany/4/1969	CY021837
1969	Bilthoven/17938/1969	CY113053
1969	Bilthoven/808/1969	CY112257
1969	Bilthoven/908/1969	CY113045
1969	Hong Kong/3/1969	CY006299
1970	Albany/1/1970	CY022938
1970	Albany/2/1970	CY021085
1970	Albany/3/1970	CY022946
1970	Albany/6/1970	CY021117
1970	Bilthoven/2668/1970	CY112273
1970	Bilthoven/93/1970	CY112265
1971	Bilthoven/21801/1971	CY113069
1971	Bilthoven/6449/1971	CY112281
1971	Hong Kong/107/1971	CY112289
1971	Hong Kong/46/1971	CY006683
1971	Memphis/1/1971	CY002496
1971	Memphis/3/1971	CY021597
1972	Bilthoven/21793/1972	CY077820
1972	Bilthoven/23290/1972	CY113077
1972	England/42/1972	CY113085
1972	Guandong/243/1972	CY007971
1972	Hong Kong/50/1972	CY006307
1972	Hong Kong/6/1972	CY003552

NA

Year	Name	No
1968	Hong Kong/1/1968	AF348184
1969	Albany/1/1969	CY019901
1969	Albany/3/1969	CY019917
1969	Albany/4/1969	CY021839
1969	Bilthoven/808/1969	CY112259
1969	Bilthoven/908/1969	CY113047
1969	England/878/1969	AY210120
1969	Hong Kong/3/1969	CY006301
1969	Rio/6/1969	AY210121
1970	Albany/1/1970	CY022940
1970	Albany/2/1970	CY021087
1970	Albany/3/1970	CY022948
1970	Albany/6/1970	CY021119
1970	Bilthoven/2668/1970	CY077842
1970	Bilthoven/93/1970	CY112267
1970	Canada/2/1970	AY210124
1970	Queensland/7/1970	AY210125
1970	Taiwan/2/1970	AY210126
1970	Trinidad/697/1970	AY210127
1971	Bilthoven/21801/1971	CY113071
1971	Bilthoven/6449/1971	CY077862
1971	Caracas/1/1971	AY210128
1971	Chiba/5/1971	AY210129
1971	Hong Kong/107/1971	CY112291
1971	Hong Kong/46/1971	CY006685
1971	Memphis/1/1971	CY002498

1972	Memphis/101/1972	CY008676	1971	Memphis/3/1971	CY021599
1972	Memphis/102/1972	CY002096	1971	Taiwan/3/1971	AY210131
1972	Memphis/103/1972	CY008460	1972	Udorn/307/1972	DQ508931
1972	Memphis/109/1972	CY002744	1972	Bilthoven/21793/1972	CY112307
1972	Udorn/307/1972	DQ508929	1972	Bilthoven/23290/1972	CY113079
1973	Bilthoven/3517/1973	CY113101	1972	England/42/1972	AY210132
1973	Bilthoven/552/1973	CY113093	1972	Guandong/243/1972	CY007973
1973	Bilthoven/748/1973	CY112321	1972	Hong Kong/6/1972	CY003554
1973	Hong Kong/11/1973	CY003528	1972	Memphis/101/1972	CY008678
1973	Hong Kong/33/1973	CY009004	1972	Memphis/102/1972	AB124659
1973	Memphis/3/1973	CY006811	1972	Memphis/103/1972	CY008462
1973	Port Chalmers/1/1973	CY009348	1972	Memphis/109/1972	CY002746
1973	Port Chalmers/JY2/1973	CY147430	1972	PuertoRico/3/1972	AY210133
1974	Albany/20/1974	CY021093	1972	Taiwan/1/1972	AY210134
1974	Bilthoven/5146/1974	CY112329	1972	Tokyo/31/1972	AY210135
1974	Bilthoven/5930/1974	CY113117	1973	Bilthoven/3517/1973	CY113103
1974	Bilthoven/5931/1974	CY113125	1973	Bilthoven/552/1973	CY113095
1974	HongKong/14/1974	CY003496	1973	Bilthoven/748/1973	CY112323
1974	HongKong/49/1974	CY006907	1973	Hong Kong/11/1973	CY003530
1974	Memphis/101/1974	CY006715	1973	Hong Kong/33/1973	CY009006
1974	Memphis/102/1974	CY006819	1973	Memphis/3/1973	CY006813
1974	Memphis/103/1974	CY006827	1973	Port Chalmers/1/1973	CY009350
1975	Albany/42/1975	CY021077	1973	Port Chalmers/JY2/1973	CY147432
1975	Beijing/39/1975	CY006044	1973	Tokyo/6/1973	AB124660
1975	Bilthoven/1843/1975	CY113157	1974	Albany/20/1974	CY021095
1975	Bilthoven/2600/1975	CY113165	1974	Bilthoven/5146/1974	CY112331
1975	Bilthoven/2813/1975	CY113173	1974	Bilthoven/5930/1974	CY113119
1975	Bilthoven/334/1975	CY113149	1974	Bilthoven/9459/1974	CY113143
1975	Bilthoven/4273/1975	CY112337	1974	Hong Kong/14/1974	CY003498
1975	Bilthoven/9459/1974	CY113141	1974	Hong Kong/49/1974	CY006909

1975	HongKong/43/1975	CY003728	1974	Memphis/101/1974	CY006717
1975	Victoria/3/1975	CY113181	1974	Memphis/102/1974	CY006821
1976	Albany/1/1976	CY021941	1975	Albany/42/1975	CY021079
1976	Albany/15/1976	CY021829	1975	Beijing/39/1975	CY006046
1976	Bilthoven/1761/1976	CY077793	1975	Bilthoven/1843/1975	CY113159
1976	Bilthoven/5657/1976	CY113213	1975	Bilthoven/2600/1975	CY113167
1976	Memphis/103/1976	CY006883	1975	Bilthoven/2813/1975	CY113175
1976	Memphis/104/1976	CY022309	1975	Bilthoven/334/1975	CY113151
1976	Memphis/106/1976	CY008692	1975	Bilthoven/4273/1975	CY112339
1976	Memphis/108/1976	CY008700	1975	Hong Kong/43/1975	CY003730
1977	Albany/4/1977	CY021101	1975	Victoria/3/1975	CY113183
1977	Amsterdam/1609/1977	CY113229	1976	Albany/1/1976	CY021943
1977	Bilthoven/3895/1977	CY113237	1976	Albany/15/1976	CY021831
1977	England/321/1977	X05907	1976	Bilthoven/1761/1976	CY113199
1977	Memphis/1/1977	CY006731	1976	Bilthoven/2271/1976	CY116574
1977	Memphis/2/1977	CY008115	1976	Bilthoven/5657/1976	CY113215
1977	Memphis/4/1977	CY008123	1976	Kumamoto/55/1976	AB124661
1977	Nanjing/49/1977	CY006763	1976	Memphis/103/1976	CY006885
1977	Rotterdam/5828/1977	CY113245	1976	Memphis/104/1976	CY022311
1977	Rotterdam/8179/1977	CY113253	1976	Memphis/106/1976	CY008694
1977	Texas/1/1977	CY113261	1976	Memphis/108/1976	CY008702
1978	Albany/14/1978	CY020221	1977	Albany/4/1977	CY021103
1978	Memphis/12/1978	CY006699	1977	Amsterdam/1609/1977	CY113231
1978	Memphis/16/1978	CY020325	1977	Bilthoven/3895/1977	CY113239
1978	Memphis/18/1978	CY006707	1977	Memphis/1/1977	CY006733
1979	Bangkok/1/1979	CY114429	1977	Memphis/2/1977	CY008117
1980	HongKong/45/1980	CY006043	1977	Memphis/4/1977	CY008125
1980	HongKong/46/1980	CY003488	1977	Rotterdam/5828/1977	CY113247
1980	Memphis/1/1980	CY008660	1977	Rotterdam/8179/1977	CY113255
1980	Memphis/3/1980	CY008468	1977	Texas/1/1977	AB124662

1980	Memphis/4/1980	CY007619	1977	Yamanashi/2/77	AB124663
1980	Memphis/9/1980	CY006891	1978	Albany/14/1978	CY020223
1980	Nanjing/13/1980	CY006203	1978	Memphis/12/1978	CY006701
1980	Netherlands/209/1980	CY113269	1978	Memphis/16/1978	CY020327
1980	Rotterdam/577/1980	CY112353	1978	Memphis/18/1978	CY006709
1981	Bilthoven/4791/1981	CY112361	1979	Bangkok/1/1979	K01150
1981	Lyon/2380/1981	CY113277	1980	Hong Kong/45/1980	CY006101
1981	Memphis/1/1981	CY007627	1980	Hong Kong/46/1980	CY003490
1982	HongKong/1/1982	CY006052	1980	Memphis/1/1980	CY008662
1982	Nanjing/2/1982	CY006755	1980	Memphis/3/1980	CY008470
1982	Netherlands/233/1982	CY114437	1980	Memphis/9/1980	CY006893
1982	Netherlands/241/1982	CY113293	1980	Nanjing/13/1980	CY006205
1982	Philippines/2/1982	CY113301	1980	Netherlands/209/1980	CY113271
1982	Philippines/2-MA/1982	CY065976	1980	Rotterdam/577/1980	CY112355
1983	HongKong/14/1983	CY006315	1981	Bilthoven/4791/1981	CY112363
1983	HongKong/26/1983	CY003720	1981	Lyon/2380/1981	CY113279
1983	HongKong/5/1983	CY003736	1981	Memphis/1/1981	CY007629
1983	Memphis/33/1983	CY009052	1982	Bilthoven/10684/1982	CY113287
1983	Nanjing/36/1983	CY006851	1982	Hong Kong/1/1982	CY006054
1983	Oslo/13676/1983	CY113309	1982	Nanjing/2/1982	CY006757
1984	Caen/1/1984	CY187384	1982	Netherlands/233/1982	CY114439
1984	HongKong/4/1984	CY006323	1982	Netherlands/241/1982	CY113295
1984	HongKong/7/1984	CY003744	1982	Philippines/2/1982	CY065970
1984	Nanjing/28/1984	CY008172	1982	Philippines/2-MA/1982	CY065978
1985	Guildford/V728/1985	CY113317	1983	Hong Kong/14/1983	CY006317
1985	HongKong/24/1985	CY003520	1983	Hong Kong/26/1983	CY003722
1985	HongKong/6/1985	CY003504	1983	Hong Kong/5/1983	CY003738
1985	HongKong/7/1985	CY003536	1983	Memphis/33/1983	CY009054
1985	Memphis/2/1985	CY009068	1983	Nanjing/36/1983	CY006853
1985	Memphis/5/1985	CY008452	1983	Oslo/13676/1983	CY113311

1985	Memphis/7/1985	CY008708	1984	Caen/1/1984	CY116580
1985	Netherlands/330/1985	CY113325	1984	Hong Kong/4/1984	CY006325
1985	Netherlands/333/1985	CY113333	1984	Hong Kong/7/1984	CY003746
1985	Stockholm/10/1985	CY113341	1984	Nanjing/28/1984	CY008174
1985	Wellington/4/1985	CY113349	1985	Guildford/V728/1985	CY113319
1986	Colorado/2/1986	CY113357	1985	Hong Kong/24/1985	CY003522
1986	Leningrad/360/1986	DQ508849	1985	Hong Kong/6/1985	CY003506
1986	Memphis/1/1986	CY002752	1985	Hong Kong/7/1985	CY003538
1986	Memphis/11/1986	CY008716	1985	Memphis/12/1985	CY011474
1987	HongKong/7/1987	CY003544	1985	Memphis/5/1985	CY008454
1987	Shanghai/11/1987	CY113365	1985	Memphis/7/1985	CY008710
1987	Sichuan/2/1987	CY112396	1985	Netherlands/330/1985	CY113327
1988	Atlanta/3572/1988	CY113373	1985	Netherlands/333/1985	CY113335
1988	Berlin/6/1988	AJ252129	1985	Stockholm/10/1985	CY113343
1988	Cottbus/42/1988	AJ252131	1985	Wellington/4/1985	CY113351
1988	England/427/1988	CY113381	1986	Colorado/2/1986	CY113359
1988	HongKong/2/1988	CY003512	1986	Leningrad/360/1986	DQ508851
1988	Memphis/15/1988	CY008732	1986	Memphis/1/1986	CY002754
1988	Memphis/5/1988	CY008724	1986	Memphis/11/1986	CY008718
1988	Netherlands/450/1988	CY113389	1987	Hong Kong/7/1987	CY003546
1988	Oklahoma/5/1988	CY114453	1987	Shanghai/11/1987	U42633
1988	Siena/3/1988	CY035198	1987	Sichuan/2/1987	CY112398
1988	Stockholm/12/1988	CY113397	1988	Atlanta/3572/1988	CY113375
1988	Victoria/1/1988	CY114445	1988	Christchurch/2/1988	CY116579
1989	Atlanta/211/1989	CY113405	1988	England/427/1988	CY113383
1989	Beijing/352/1989	CY112404	1988	Hong Kong/2/1988	CY003514
1989	Beijing/353/1989	CY114461	1988	Memphis/15/1988	CY008734
1989	Eindhoven/3447/1989	CY112412	1988	Memphis/5/1988	CY008726
1989	England/138/1989	CY113413	1988	Netherlands/450/1988	CY113391
1989	Geneva/5007/1989	CY112420	1988	Oklahoma/5/1988	CY114455

1989	HongKong/1/1989	CY112428
1989	Netherlands/620/1989	KM821283
1989	Singapore/34/1989	CY113453
1989	Singapore/53/1989	CY112436
1989	Victoria/1/1989	CY113477
1989	Waikato/1/1989	CY113493
1989	Wellington/5/1989	CY113485
1990	Memphis/1/1990	CY003064
1990	Memphis/2/1990	CY011480
1990	Memphis/5/1990	CY113509
1990	Memphis/7/1990	CY008740
1990	Shanghai/24/1990	CY113517
1990	Siena/10/1990	CY035214
1990	Siena/4/1990	CY035206
1990	Suita/1/1990	CY113525
1990	Victoria/2/1990	CY113533
1990	Wellington/3/1990	CY113541
1991	Canberra/1/1991	CY113549
1991	England/260/1991	CY113557
1991	Geneva/5366/1991	CY113565
1991	Lyon/1149/1991	CY113573
1991	Lyon/1182/1991	CY113581
1991	Madrid/G12/1991	CY113653
1991	Netherlands/816/1991	CY113661
1991	Siena/3/1991	CY043728
1991	Stockholm/20/1991	CY112452
1992	Amsterdam/4112/1992	CY112460
1992	Beijing/32/1992	U26830
1992	Finland/218/1992	CY112476
1992	Finland/220/1992	CY112484
1988	Siena/3/1988	CY035200
1988	Stockholm/12/1988	CY113399
1988	Victoria/1/1988	CY114447
1989	Atlanta/211/1989	CY113407
1989	Beijing/352/1989	CY112406
1989	Beijing/353/1989	DQ508835
1989	Eindhoven/3447/1989	CY116582
1989	England/138/1989	CY113415
1989	Geneva/5007/1989	CY112422
1989	Hong Kong/1/1989	CY112430
1989	Netherlands/620/1989	CY113423
1989	Singapore/34/1989	CY113455
1989	Singapore/53/1989	CY112438
1989	Victoria/1/1989	CY113479
1989	Waikato/1/1989	CY113495
1989	Wellington/5/1989	CY113487
1990	Memphis/1/1990	CY003066
1990	Memphis/2/1990	CY113503
1990	Memphis/5/1990	CY113511
1990	Memphis/7/1990	CY008742
1990	Shanghai/24/1990	CY113519
1990	Siena/10/1990	CY035216
1990	Siena/4/1990	CY035208
1990	Victoria/2/1990	CY113535
1990	Wellington/3/1990	CY113543
1991	Canberra/1/1991	CY113551
1991	England/260/1991	CY113559
1991	Geneva/5366/1991	CY113567
1991	Lyon/1149/1991	CY113575
1991	Lyon/1182/1991	CY113583

1992	Geneva/5113/1992	CY113685
1992	Harbin/15/1992	CY121325
1992	HongKong/14/1992	CY003712
1992	Houston/56798/1992	CY113693
1992	Houston/56829/1992	CY113701
1992	Madrid/G58/1992	CY113717
1992	Rotterdam/100540/1992	CY112508
1992	Sendai/C273/1992	CY112516
1992	SouthAustralia/23/1992	CY113885
1992	SouthAustralia/27/1992	CY113893
1992	Stockholm/13/1992	CY113917
1993	Akita/4/1993	CY113957
1993	Guangdong/25/1993	CY113973
1993	Lyon/672/1993	CY113981
1993	Madrid/G102/1993	CY112564
1993	Netherlands/17/1993	CY112589
1993	NewYork/779/1993	CY012776
1993	NewYork/793/1993	CY014143
1993	NewYork/800/1993	CY016483
1993	Oslo/2219/1993	CY112637
1993	Paris/287/1993	CY114093
1993	Shandong/9/1993	KM821294
1993	Shiga/6/1993	CY112653
1993	Singapore/19/1993	CY112661
1993	Stockholm/20/1993	CY112669
1993	Victoria/104/1993	CY112677
1993	Wellington/59/1993	CY112685
1994	England/7/1994	CY112701
1994	HongKong/1/1994	CY112709
1994	HongKong/55/1994	CY112725
1991	Madrid/G12/1991	CY113655
1991	Netherlands/816/1991	CY113663
1991	Siena/3/1991	CY043730
1991	Stockholm/20/1991	CY112454
1992	Amsterdam/4112/1992	CY112462
1992	Beijing/32/1992	CY033608
1992	Finland/218/1992	CY112478
1992	Finland/220/1992	CY112486
1992	Geneva/5113/1992	CY113687
1992	Harbin/15/1992	U42773
1992	Hong Kong/14/1992	CY003714
1992	Houston/56798/1992	CY113695
1992	Houston/56941/1992	CY113711
1992	Madrid/G58/1992	CY113719
1992	Rotterdam/100540/1992	CY112510
1992	Sendai/C273/1992	CY112518
1992	South Australia/23/1992	CY113887
1992	South Australia/27/1992	CY113895
1992	Stockholm/13/1992	CY113919
1993	Akita/4/1993	CY113959
1993	Guangdong/25/93	U42778
1993	Lyon/672/1993	CY113983
1993	Madrid/G102/1993	CY112567
1993	Nanchang/58/1993	CY006349
1993	Netherlands/165/1993	CY114063
1993	Netherlands/17/1993	CY112591
1993	New York/779/1993	CY012778
1993	New York/793/1993	CY014145
1993	New York/800/1993	CY016485
1993	Oslo/2219/1993	CY112639

1994	Johannesburg/33/1994	KM821293
1994	Nanchang/0058/1994	CY006339
1994	Nanchang/0074/1994	CY003752
1994	Netherlands/18/1994	CY114157
1994	NewYork/717/1994	CY013887
1994	NewYork/733/1994	CY010988
1994	NewYork/742/1994	CY013343
1994	SouthAustralia/15/1994	CY112749
1995	Finland/338/1995	CY114165
1995	HongKong/32/1995	CY112765
1995	Lyon/2279/1995	CY114213
1995	Malaysia/07145/1995	CY118418
1995	Memphis/24/1995	CY002272
1995	Nanchang/933/1995	KM821307
1995	Netherlands/1/1995	CY114221
1995	NewYork/606/1995	CY010620
1995	NewYork/700/1995	CY012520
1995	Siena/3/1995	CY038503
1995	Stockholm/5/1995	CY112805
1995	Victoria/75/1995	CY112813
1995	Wuhan/359/1995	KM821302
1996	Brisbane/8/1996	CY114477
1996	Geneva/3958/1996	CY114229
1996	HongKong/20/1996	CY114237
1996	Lyon/1781/1996	CY114277
1996	Malaysia/10081/1996	CY117698
1996	Netherlands/91/1996	CY114285
1996	NewYork/554/1996	CY012848
1996	NewYork/592/1996	CY010036
1996	NewYork/637/1996	CY010732
1993	Paris/287/1993	CY114095
1993	Shandong/9/1993	U43419
1993	Shiga/6/1993	CY112655
1993	Singapore/19/1993	CY112663
1993	Stockholm/20/1993	CY112671
1993	Victoria/104/1993	CY112679
1993	Wellington/59/1993	CY112687
1994	England/7/1994	CY112703
1994	Hong Kong/1/1994	CY112711
1994	Hong Kong/55/1994	CY112727
1994	Johannesburg/33/1994	CY112735
1994	Nanchang/0074/1994	CY003754
1994	Netherlands/18/1994	CY114159
1994	New York/717/1994	CY013889
1994	New York/733/1994	CY010990
1994	New York/742/1994	CY013345
1994	South Australia/15/1994	CY112751
1995	Buenos Aires/4084/95	AF533993
1995	Finland/338/1995	CY114167
1995	Hong Kong/32/1995	CY112767
1995	Lyon/2279/1995	CY114215
1995	Malaysia/07145/1995	CY118420
1995	Memphis/24/1995	CY002274
1995	Nanchang/933/1995	AJ457945
1995	Netherlands/1/1995	CY114223
1995	New York/606/1995	CY010622
1995	New York/678/1995	CY012506
1995	New York/700/1995	CY012522
1995	Siena/3/1995	CY038505
1995	Stockholm/5/1995	CY112807

1996	Nice/491/1996	CY112861	1995	Victoria/75/1995	CY112815
1996	Singapore/1/1996	CY112829	1995	Wuhan/359/1995	U51246
1996	SouthAfrica/1147/1996	CY121357	1996	Brisbane/8/1996	CY114479
1997	Auckland/10/1997	CY114293	1996	Geneva/3958/1996	CY114231
1997	HongKong/1/1997	CY112837	1996	Hong Kong/20/1996	CY114239
1997	HongKong/CUHK20992/1997	EU856902	1996	Lyon/1781/1996	CY114279
1997	Johannesburg/10/1997	CY112853	1996	Malaysia/10081/1996	CY117700
1997	Malaysia/13241/1997	CY117752	1996	Netherlands/91/1996	CY114287
1997	Netherlands/300/1997	CY114301	1996	New York/554/1996	CY012850
1997	NewYork/501/1997	CY006443	1996	New York/592/1996	CY010038
1997	NewYork/508/1997	CY006243	1996	New York/637/1996	CY010734
1997	NewYork/583/1997	CY009524	1996	Nice/491/1996	CY112863
1997	Oslo/244/1997	CY112645	1996	South Africa/1147/1996	CY121359
1997	Siena/1/1997	CY036847	1997	Auckland/10/1997	CY114295
1997	Sydney/5/1997	CY112885	1997	Hong Kong/1/1997	CY112839
1998	HongKong/CUHK10186/1998	EU856819	1997	Hong Kong/CUHK20992/1997	EU857183
1998	HongKong/CUHK10660/1998	EU856827	1997	Johannesburg/10/1997	CY112855
1998	Malaysia/17230/1998	CY118602	1997	Malaysia/13241/1997	CY117754
1998	Memphis/31/1998	AY271794	1997	Netherlands/300/1997	CY114303
1998	Netherlands/427/1998	CY112901	1997	New York/501/1997	CY006445
1998	NewYork/224/1998	CY001568	1997	New York/508/1997	CY006245
1998	NewYork/550/1998	CY008980	1997	New York/583/1997	CY009526
1998	Switzerland/7729/1998	AY032978	1997	Oslo/244/1997	CY112647
1998	Thailand/Siriraj-08/1998	EF568929	1997	Siena/1/1997	CY036849
1999	California/32/1999	CY121424	1997	Sydney/5/1997	AJ291403
1999	Canterbury/179/1999	CY009108	1998	Hong Kong/CUHK10186/1998	EU857100
1999	Georgia/NHRC0001/1999	CY090885	1998	Hong Kong/CUHK10660/1998	EU857108
1999	Hong Kong/1143/1999	AF382319	1998	Malaysia/17230/1998	CY118604
1999	Hong Kong/1180/1999	AF382325	1998	Memphis/31/1998	AY271795
1999	Hong Kong/CUHK30423/1999	EU856967	1998	Netherlands/427/1998	CY112903

1999	Illinois/NHRC0001/1999	CY090893
1999	Malaysia/10717/1999	CY118474
1999	Memphis/59/1999	CY002112
1999	Moscow/10/1999	CY112909
1999	Netherlands/301/1999	CY114493
1999	NewSouthWales/15/1999	CY016539
1999	NewYork/141/1999	CY000801
1999	Panama/2007/1999	KM821317
1999	SouthCarolina/NHRC0001/1999	CY090901
2000	Auckland/583/2000	CY025018
2000	Canterbury/2/2000	CY009116
2000	Denmark/203/2000	EU103728
2000	Dunedin/1/2000	CY012616
2000	HongKong/CUHK20300/2000	EU856898
2000	HongKong/CUHK21128/2000	EU856904
2000	HongKong/CUHK26969/2000	EU856954
2000	Hutt/82/2000	CY008812
2000	Malaysia/14590/2000	CY117776
2000	Missouri/NHRC0001/2000	CY090909
2000	Netherlands/3/2000	CY114317
2000	NewSouthWales/27/2000	CY016092
2000	NewYork/142/2000	CY001520
2000	NewYork/180/2000	CY000737
2000	NewYork/436/2000	CY003264
2000	UlanUde/01/2000	CY121408
2000	WesternAustralia/1/2000	CY017323
2001	Auckland/602/2001	CY022525
2001	Canterbury/10/2001	CY009396
2001	HaNoi/1766/2001	CY105190
2001	HongKong/CUHK13048/2001	EU856853

1998	New York/224/1998	CY001570
1998	New York/550/1998	CY008982
1998	Thailand/Siriraj-08/1998	JN617985
1999	California/32/1999	CY121426
1999	Canterbury/179/1999	CY009110
1999	Georgia/NHRC0001/1999	CY090887
1999	Hong_Kong/1143/1999	AF382329
1999	Hong_Kong/1180/1999	AF386763
1999	Hong_Kong/CUHK30423/1999	EU857248
1999	Illinois/NHRC0001/1999	CY090895
1999	Malaysia/10717/1999	CY118476
1999	Memphis/59/1999	CY002114
1999	Moscow/10/1999	DQ487331
1999	Netherlands/301/1999	CY114495
1999	New_South_Wales/15/1999	CY016541
1999	Panama/2007/1999	CY034102
1999	South_Carolina/NHRC0001/1999	CY090903
2000	Auckland/583/2000	CY025020
2000	Canterbury/2/2000	CY009118
2000	Denmark/203/2000	EU103906
2000	Dunedin/1/2000	CY012618
2000	Hong_Kong/CUHK20300/2000	EU857179
2000	Hong_Kong/CUHK21128/2000	EU857185
2000	Hong_Kong/CUHK26969/2000	EU857235
2000	Hutt/82/2000	CY008814
2000	Malaysia/14590/2000	CY117778
2000	Missouri/NHRC0001/2000	CY090911
2000	Netherlands/3/2000	CY114319
2000	New_South_Wales/27/2000	CY016094
2000	New_York/142/2000	CY001522

2001	HongKong/CUHK21421/2001	EU856910	2000	New_York/180/2000	CY000739
2001	Malaysia/17998/2001	CY118626	2000	New_York/436/2000	CY003266
2001	Netherlands/118/2001	CY112925	2000	Ulan_Ude/01/2000	CY121410
2001	NewYork/127/2001	CY000584	2000	Western_Australia/1/2000	CY017325
2001	NewYork/402/2001	CY003088	2001	Auckland/602/2001	CY022527
2001	NewYork/84/2001	CY000201	2001	Canterbury/10/2001	CY009398
2001	Queensland/11/2001	CY017483	2001	HaNoi/1766/2001	CY105192
2002	Auckland/608/2002	CY022549	2001	Hong_Kong/CUHK13048/2001	EU857134
2002	Canterbury/02/2002	CY007595	2001	Hong_Kong/CUHK21421/2001	EU857191
2002	Canterbury/80/2002	CY007771	2001	Malaysia/17998/2001	CY118628
2002	Denmark/01/2002	EU103631	2001	Netherlands/118/2001	CY112927
2002	Dunedin/10/2002	CY011400	2001	New_York/127/2001	CY000578
2002	Fujian/411/2002	KM821324	2001	New_York/84/2001	CY000203
2002	Genoa/1/2002	CY037343	2001	Queensland/11/2001	CY017485
2002	Georgia/NHRC0001/2002	CY091109	2002	Auckland/608/2002	CY022551
2002	HongKong/CUHK13249/2002	EU856857	2002	Canterbury/02/2002	CY007597
2002	Illinois/NHRC0001/2002	CY090917	2002	Canterbury/80/2002	CY007773
2002	Malaysia/23606/2002	CY118658	2002	Denmark/01/2002	EU103825
2002	Missouri/NHRC0001/2002	CY091093	2002	Dunedin/10/2002	CY011402
2002	Morioka/52/2002	AB434108	2002	Fujian/411/2002	CY088485
2002	Netherlands/120/2002	CY114341	2002	Genoa/1/2002	CY037345
2002	NewYork/101/2002	CY001104	2002	Georgia/NHRC0001/2002	CY091111
2002	NewYork/92/2002	CY000289	2002	Hong_Kong/CUHK13249/2002	EU857138
2002	Queensland/21/2002	CY017531	2002	Illinois/NHRC0001/2002	CY090919
2002	Texas/NHRC0001/2002	CY091101	2002	Malaysia/23606/2002	CY118660
2002	Waikato/23/2002	CY015556	2002	Missouri/NHRC0001/2002	CY091095
2003	Australia/NHRC0001/2003	CY091181	2002	Morioka/52/2002	AB433819
2003	California/NHRC0006/2003	CY091461	2002	Netherlands/120/2002	CY114343
2003	Canterbury/417/2003	CY007107	2002	New_York/101/2002	CY001106
2003	England/516/2003	CY088198	2002	New_York/92/2002	CY000291

2003	Finland/170/2003	CY114349
2003	Fujian/445/2003	CY121448
2003	Genoa/14/2003	CY037351
2003	HaNoi/ARI189/2003	CY104076
2003	Malaysia/26227/2003	CY117980
2003	Mexico/InDRE2662/2003	CY100594
2003	Moscow/346/2003	DQ089638
2003	Netherlands/213/2003	KM821321
2003	NewYork/61A/2003	CY000001
2003	Oklahoma/323/03	DQ059385
2003	Queensland/33/2003	CY017563
2003	Scotland/79/2003	CY088142
2003	Singapore/NHRC0001/2003	CY091149
2003	SouthCarolina/NHRC0002/2003	CY090949
2003	Thailand/Siriraj-02/2003	EF568926
2003	TW/229/03	DQ415322
2003	Waikato/154/2003	CY012072
2003	WesternAustralia/37/2003	CY015772
2003	Wyoming/03/2003	EU268227
2003	Yokohama/2013/2003	AB671293
2004	California/7/2004	CY114373
2004	Canterbury/17/2004	CY007363
2004	Christchurch/13/2004	CY002906
2004	Denmark/1-2/2004	EU103714
2004	Georgia/NHRC0001/2004	CY090973
2004	HaNoi/HN30110/2004	CY104140
2004	HongKong/CUHK-22910/2004	EU516331
2004	HongKong/HKU2/2004	CY038567
2004	HongKong/HKU40/2004	CY039047
2004	Hyogo/36/2004	AB761206

2002	Queensland/21/2002	CY017533
2002	Texas/NHRC0001/2002	CY091103
2002	Waikato/23/2002	CY015558
2003	Australia/NHRC0001/2003	CY091183
2003	California/NHRC0006/2003	CY091463
2003	Canterbury/417/2003	CY007109
2003	Denmark/73/2003	EU103944
2003	England/516/2003	CY088200
2003	Finland/170/2003	CY114351
2003	Fujian/445/2003	CY121450
2003	Genoa/14/2003	CY037353
2003	HaNoi/ARI189/2003	CY104078
2003	Malaysia/26227/2003	CY117982
2003	Mexico/InDRE2662/2003	CY100596
2003	Netherlands/213/2003	CY001407
2003	New_York/61A/2003	CY000003
2003	Oklahoma/323/2003	DQ059384
2003	Queensland/33/2003	CY017565
2003	Scotland/79/2003	CY088144
2003	Singapore/NHRC0001/2003	CY091151
2003	South_Carolina/NHRC0002/2003	CY090951
2003	Thailand/Siriraj-02/2003	JN617983
2003	TW/229/2003	DQ415344
2003	Waikato/154/2003	CY012074
2003	Western_Australia/37/2003	CY015774
2003	Wyoming/03/2003	AY531034
2003	Yokohama/2013/2003	AB671294
2004	California/7/2004	EF541462
2004	Canterbury/17/2004	CY007365
2004	Christchurch/13/2004	CY002908

2004	Illinois/NHRC0001/2004	CY092108	2004	Denmark/1-2/2004	EU103890
2004	Malaysia/26822/2004	CY118730	2004	Georgia/NHRC0001/2004	CY090975
2004	Missouri/NHRC0002/2004	CY091117	2004	HaNoi/HN30110/2004	CY104142
2004	NewYork/377/2004	CY002592	2004	Hong_Kong/CUHK-22910/2004	EU516332
2004	NewYork/392/2004	CY002064	2004	Hong_Kong/HKU2/2004	CY038569
2004	NewYork/472/2004	CY008164	2004	Hong_Kong/HKU40/2004	CY039049
2004	Queensland/47/2004	CY017587	2004	Hyogo/36/2004	AB761208
2004	Taiwan/9/2004	CY040082	2004	Illinois/NHRC0001/2004	CY092110
2004	Thailand/Siriraj-03/2004	EF568924	2004	Malaysia/26822/2004	CY118732
2004	Wellington/01/2004	CY121480	2004	Missouri/NHRC0002/2004	CY091119
2004	WesternAustralia/51/2004	CY015876	2004	New_York/377/2004	CY002594
2005	Auckland/616/2005	CY022573	2004	New_York/392/2004	CY002066
2005	Australia/NHRC0001/2005	CY091421	2004	Queensland/47/2004	CY017589
2005	California/NHRC0005/2005	CY091517	2004	Taiwan/9/2004	CY040084
2005	Canterbury/124/2005	CY009044	2004	Thailand/Siriraj-03/2004	JN617986
2005	Denmark/200/2005	EU103761	2004	Wellington/1/2004	EF512559
2005	Georgia/NHRC0001/2005	CY091125	2004	Western_Australia/51/2004	CY015878
2005	Guangdong/03/2005	EU604298	2005	Auckland/616/2005	CY022575
2005	Guangdong/04/2005	EU620747	2005	Australia/NHRC0001/2005	CY091423
2005	Guangdong/05/2005	EU620755	2005	California/NHRC0005/2005	CY091519
2005	Guangdong/07/2005	EU620763	2005	Canterbury/124/2005	CY009046
2005	Guangdong/09/2005	EU620771	2005	Denmark/200/2005	EU103931
2005	HaNoi/311/2005	CY105886	2005	Georgia/NHRC0001/2005	CY091127
2005	HongKong/CUHK10026/2005	EU856814	2005	Guangdong/03/2005	EU604299
2005	Illinois/NHRC0001/2005	CY091005	2005	Guangdong/04/2005	EU620748
2005	Malaysia/31944/2005	CY118826	2005	Guangdong/05/2005	EU620756
2005	Mexico/DIF2246/2005	KJ855419	2005	Guangdong/07/2005	EU620764
2005	Morioka/35/2005	AB434109	2005	Guangdong/09/2005	EU620772
2005	Netherlands/548/2005	CY112997	2005	HaNoi/311/2005	CY105888
2005	NewYork/210/2005	CY002776	2005	Hong_Kong/CUHK10026/2005	EU857095

2005	Ontario/RV1273/2005	DQ469962
2005	Queensland/54/2005	CY019021
2005	SouthCarolina/NHRC0001/2005	CY090989
2005	Texas/NHRC0001/2005	CY091133
2005	Wisconsin/67/2005	KM821341
2006	BRISBANE/9/2006	CY121568
2006	California/NHRC0001/2006	CY091053
2006	Denmark/10/2006	EU103762
2006	Denmark/27/2006	EU103673
2006	Georgia/NHRC0001/2006	CY091141
2006	HongKong/CUHK53005/2006	EU857049
2006	Illinois/NHRC0001/2006	CY091045
2006	Malaysia/1657167/2006	CY118906
2006	Mexico/DIF29/2006	KJ855363
2006	Missouri/NHRC0001/2006	CY091077
2006	Morioka/17/2006	AB434111
2006	Nepal/921/2006	CY121512
2006	Netherlands/363/2006	CY114397
2006	NewYork/1005/2006	CY172207
2006	Ohio/06/2006	EU516029
2006	SouthCarolina/NHRC0001/2006	CY091061
2006	Texas/NHRC0002/2006	CY091069
2006	Thailand/CU124/2006	EU021284
2006	Wisconsin/45/2006	EU100715
2007	Alabama/UR06-0482/2007	CY026035
2007	Boston/14/2007	CY044492
2007	Brisbane/10/2007	KM978060
2007	California/UR06-0347/2007	CY025421
2007	Cheongju/H396/2007	FJ009470
2007	Colorado/UR06-0535/2007	CY026163

2005	Illinois/NHRC0001/2005	CY091007
2005	Malaysia/31944/2005	CY118828
2005	Mexico/DIF2246/2005	CY100580
2005	Morioka/35/2005	AB433820
2005	Netherlands/548/2005	CY112999
2005	New_York/210/2005	CY002778
2005	Ontario/RV1273/2005	DQ469960
2005	Queensland/54/2005	CY019023
2005	South_Carolina/NHRC0001/2005	CY090991
2005	Texas/NHRC0001/2005	CY091135
2005	Wisconsin/67/2005	EF541463
2006	Brisbane/09/2006	CY121570
2006	California/NHRC0001/2006	CY091055
2006	Denmark/10/2006	EU103932
2006	Denmark/27/2006	EU103853
2006	Georgia/NHRC0001/2006	CY091143
2006	Hong_Kong/CUHK53005/2006	EU857330
2006	Illinois/NHRC0001/2006	CY091047
2006	Malaysia/1657167/2006	CY118908
2006	Mexico/DIF29/2006	KJ855365
2006	Missouri/NHRC0001/2006	CY091079
2006	Morioka/17/2006	AB433822
2006	Nepal/921/2006	CY121514
2006	Netherlands/363/2006	CY114399
2006	New_York/1005/2006	CY172209
2006	Ohio/06/2006	EU516190
2006	South_Carolina/NHRC0001/2006	CY091063
2006	Texas/NHRC0002/2006	CY091071
2006	Thailand/CU124/2006	EU021285
2006	Wisconsin/45/2006	EU100656

2007	Florida/UR06-0597/2007	CY025341
2007	Guangdong/04/2007	CY091825
2007	Guangdong/15/2007	CY091829
2007	Guangdong/17/2007	CY091831
2007	Guangdong/30/2007	CY091833
2007	HaNoi/GS0740/2007	CY104340
2007	Illinois/UR06-0567/2007	CY025277
2007	Kentucky/UR06-0370/2007	CY025907
2007	Malaysia/1768409/2007	CY118051
2007	Mexico/NAY2090/2007	KJ855395
2007	Netherlands/69/2007	CY114405
2007	NewYork/UR06-0515/2007	CY025485
2007	Ohio/UR06-0410/2007	CY027499
2007	Ontario/1252/2007	EU399751
2007	Oregon/UR06-0273/2007	CY027075
2007	Peru/WRAIR1508P/2007	CY070144
2007	Taiwan/70002/2007	CY039399
2007	Thailand/CU272/2007	EU021270
2007	Tokyo/Ut-Sk-1/2007	CY049748
2007	Uruguay/716/2007	EU716426
2007	Vermont/UR06-0483/2007	CY025867
2007	Virginia/UR06-0580/2007	CY027867
2007	Washington/42/2007	EU885540
2008	Cheongju/H473/2008	FJ009477
2008	DaNang/DN446/2008	CY104622
2008	Florida/10/2008	EU885501
2008	Georgia/07/2008	EU885505
2008	Guangdong/314/2008	CY091835
2008	Guangdong/548/2008	CY091851
2008	Guangdong/578/2008	CY091855
2007	Alabama/UR06-0482/2007	CY026037
2007	Boston/14/2007	CY044494
2007	Brisbane/10/2007	KM978074
2007	California/UR06-0347/2007	CY025423
2007	Cheongju/H396/2007	FJ009482
2007	Colorado/UR06-0535/2007	CY026165
2007	Florida/UR06-0597/2007	CY025343
2007	Guangdong/04/2007	CY091826
2007	Guangdong/15/2007	CY091830
2007	Guangdong/17/2007	CY091832
2007	Guangdong/30/2007	CY091834
2007	HaNoi/GS0740/2007	CY104342
2007	Illinois/UR06-0567/2007	CY025279
2007	Kentucky/UR06-0370/2007	CY025909
2007	Malaysia/1768409/2007	CY118053
2007	Mexico/NAY2090/2007	KJ855397
2007	Netherlands/69/2007	CY114407
2007	New York/UR06-0515/2007	CY025487
2007	Ohio/UR06-0410/2007	CY027501
2007	Ontario/1252/2007	EU399753
2007	Oregon/UR06-0273/2007	CY027077
2007	Peru/WRAIR1508P/2007	CY070146
2007	Taiwan/70002/2007	CY039401
2007	Texas/12/2007	EU516020
2007	Thailand/CU272/2007	EU021271
2007	Tokyo/Ut-Sk-1/2007	CY049750
2007	Uruguay/716/2007	EU716427
2007	Vermont/UR06-0483/2007	CY025869
2007	Virginia/UR06-0580/2007	CY027869
2007	Washington/42/2007	EU885541

2008	Guangdong/93/2008	CY091857	2008	Cheongju/H473/2008	FJ009489
2008	Guangdong/ST798/2008	KJ473722	2008	DaNang/DN446/2008	CY104624
2008	Indiana/05/2008	EU885507	2008	Florida/10/2008	EU885502
2008	Japan/WRAIR1059P/2008	CY069357	2008	Georgia/07/2008	EU885506
2008	Louisiana/06/2008	EU885497	2008	Guangdong/314/2008	CY091836
2008	Malaysia/1959476/2008	CY118131	2008	Guangdong/548/2008	CY091852
2008	Memphis/28/2008	EU885503	2008	Guangdong/578/2008	CY091856
2008	Mexico/UASLP-010/2008	CY073899	2008	Guangdong/93/2008	CY091858
2008	Minnesota/05/2008	EU885499	2008	Guangdong/ST798/2008	KJ473724
2008	Mississippi/04/2008	EU885542	2008	Indiana/05/2008	EU885508
2008	Netherlands/377/2008	CY113013	2008	Japan/WRAIR1059P/2008	CY069359
2008	NewJersey/15/2008	EU885516	2008	Louisiana/06/2008	EU885498
2008	NewYork/07/2008	EU885509	2008	Malaysia/1959476/2008	CY118133
2008	NewYork/09/2008	EU885538	2008	Memphis/28/2008	EU885504
2008	Penza/55/2008	JQ988027	2008	Mexico/UASLP-010/2008	CY073901
2008	Taiwan/70120/2008	CY040098	2008	Minnesota/05/2008	EU885500
2008	Tennessee/04/2008	EU885518	2008	Mississippi/04/2008	EU885543
2008	Thailand/CU370/2008	FJ912984	2008	Netherlands/377/2008	CY113015
2008	Thueringen/2202/2008	FJ183468	2008	New Jersey/15/2008	EU885517
2008	Washington/04/2008	EU885522	2008	New York/07/2008	EU885510
2009	Alaska/24/2009	KC535312	2008	New York/09/2008	EU885539
2009	Australia/19/2009	CY061898	2008	Penza/55/2008	JQ988028
2009	Australia/22/2009	CY080539	2008	Taiwan/70120/2008	CY040100
2009	Finland/97/2009	CY121752	2008	Tennessee/04/2008	EU885519
2009	Florida/22/2009	JX905406	2008	Thailand/CU370/2008	FJ912986
2009	Guangdong/1105/2009	CY091827	2008	Thueringen/2202/2008	FJ183469
2009	Guangdong/423/2009	CY091839	2008	Washington/04/2008	EU885523
2009	Guangdong/472/2009	CY091847	2009	Alaska/24/2009	KC535311
2009	Guangdong/522/2009	CY091849	2009	Australia/19/2009	CY061900
2009	Guangdong/560/2009	CY091853	2009	Australia/22/2009	CY080541

2009	HongKong/H090-720-V21/2009	CY106720
2009	Japan/WRAIR1140P/2009	CY069429
2009	Kansas/07/2009	KC535324
2009	Korea/WRAIR1038P/2009	CY069325
2009	Lipezk/225/2009	JQ988048
2009	Maryland/16/2009	KC535318
2009	Minnesota/05/2009	KC535319
2009	Montana/16/2009	KC535345
2009	Nebraska/21/2009	KC535381
2009	Netherlands/69/2009	CY113021
2009	NewYork/70/2009	KC535453
2009	Oklahoma/09/2009	KC535399
2009	Oregon/14/2009	KC535306
2009	Pennsylvania/24/2009	KC535363
2009	Perth/16/2009	GQ293081
2009	Philippines/16/2009	GQ293083
2009	Qingdao/2199/2009	CY050095
2009	Singapore/GP3303/2009	CY100075
2009	Tennessee/15/2009	KC535352
2009	Thailand/CU-B590/2009	GQ902817
2009	Vermont/21/2009	KC535375
2009	Washington/55/2009	KC535342
2009	Wisconsin/15/2009	KC535309
2010	California/10/2010	KC535428
2010	Denmark/105/2010	HQ880599
2010	Florida/11/2010	KC535302
2010	Georgia/15/2010	KC535470
2010	Guangdong/322/2010	CY091837
2010	Guangdong/429/2010	CY091841
2010	Guangdong/441/2010	CY091843
2009	Finland/97/2009	CY121754
2009	Florida/22/2009	JX905405
2009	Guangdong/1105/2009	CY091828
2009	Guangdong/423/2009	CY091840
2009	Guangdong/472/2009	CY091848
2009	Guangdong/522/2009	CY091850
2009	Guangdong/560/2009	CY091854
2009	Hong Kong/H090-720-V21/2009	CY106722
2009	Japan/WRAIR1140P/2009	CY069431
2009	Kansas/07/2009	KC535323
2009	Korea/WRAIR1038P/2009	CY069327
2009	Lipezk/225/2009	JQ988049
2009	Maryland/16/2009	KC535317
2009	Minnesota/05/2009	KC535320
2009	Montana/16/2009	KC535344
2009	Nebraska/21/2009	KC535380
2009	Netherlands/69/2009	CY113023
2009	New York/70/2009	KC535454
2009	Oklahoma/09/2009	KC535398
2009	Oregon/14/2009	KC535305
2009	Pennsylvania/24/2009	KC535362
2009	Perth/16/2009	GQ293082
2009	Philippines/16/2009	GQ293084
2009	Qingdao/2199/2009	CY050096
2009	Singapore/GP3303/2009	CY100076
2009	Tennessee/15/2009	KC535353
2009	Thailand/CU-B590/2009	GQ902819
2009	Vermont/21/2009	KC535374
2009	Washington/55/2009	KC535341
2009	Wisconsin/15/2009	KC535308

2010	Guangdong/460/2010	CY091845	2010	California/10/2010	KC535430
2010	MexicoCity/WRAIR3577T/2010	CY093511	2010	Denmark/105/2010	HQ880601
2010	Nanjing/1654/2010	HQ664924	2010	Florida/11/2010	KC535303
2010	Netherlands/034/2010	CY114509	2010	Georgia/15/2010	KC535468
2010	NewJersey/03/2010	KC882888	2010	Guangdong/322/2010	CY091838
2010	NewYork/01/2010	KC535393	2010	Guangdong/429/2010	CY091842
2010	Niigata/1149/2010	HQ703352	2010	Guangdong/441/2010	CY091844
2010	NorthCarolina/13/2010	KC882431	2010	Guangdong/460/2010	CY091846
2010	Pennsylvania/10/2010	KC535467	2010	Mexico City/WRAIR3577T/2010	CY093513
2010	Perth/10/2010	CY121496	2010	Nanjing/1654/2010	HQ664926
2010	Peru/PER261/2010	CY160281	2010	Netherlands/034/2010	CY114511
2010	Singapore/GP4453/2010	CY100113	2010	New Jersey/03/2010	KC882887
2010	Sydney/DD2-02/2010	CY090877	2010	New York/01/2010	KC535392
2010	Texas/05/2010	KC535495	2010	Niigata/1149/2010	HQ703366
2010	Thailand/CU-H1285/2010	CY074950	2010	North Carolina/13/2010	KC882432
2010	Virginia/03/2010	KC535433	2010	Pennsylvania/10/2010	KC535465
2010	Wyoming/06/2010	KC882604	2010	Perth/10/2010	CY121498
2011	Astrakhan/RII65/2011	CY114553	2010	Peru/PER261/2010	CY160283
2011	Brisbane/299/2011	KJ942792	2010	Singapore/GP4453/2010	CY100114
2011	California/05/2011	KC883183	2010	Sydney/DD2-02/2010	CY090879
2011	Denmark/22/2011	JF327386	2010	Texas/05/2010	KC535497
2011	Guangdong/94/2011	CY099953	2010	Thailand/CU-H1285/2010	CY074952
2011	Idaho/01/2011	KC882584	2010	Virginia/03/2010	KC535431
2011	Maine/05/2011	KC882526	2010	Wyoming/06/2010	KC882603
2011	Maryland/17/2011	KC882447	2011	Astrakhan/RII65/2011	CY114555
2011	Massachusetts/07/2011	KC882518	2011	Brisbane/299/2011	KJ942794
2011	Mexico/VER60/2011	KJ855339	2011	California/05/2011	KC883182
2011	Nebraska/08/2011	KC882624	2011	Denmark/22/2011	JF327388
2011	Netherlands/063/2011	CY114421	2011	Guangdong/94/2011	CY099954
2011	NewJersey/04/2011	KC882521	2011	Idaho/01/2011	KC882585

2011	Oklahoma/02/2011	KC883075
2011	Pennsylvania/12/2011	KC882450
2011	Singapore/EN122/2011	CY124151
2011	Singapore/GP1490/2011	CY124221
2011	Tennessee/F2092c90/2011	CY167317
2011	Victoria/361/2011	KJ942680
2011	Wisconsin/01/2011	KC882811
2011	Zhongshan/01/2011	KJ567654
2011	Zhongshan/02/2011	KJ567655
2012	Alabama/02/2012	KC893090
2012	Alaska/21/2012	KC892668
2012	Alborz/1095/2012	KC865653
2012	Arizona/M14/2012	KC513482
2012	Boston/DOA2-098/2012	CY148308
2012	California/07/2012	KC893075
2012	Chicago/YGA_04007/2012	CY171071
2012	Colorado/09/2012	KC892465
2012	Czech_Republic/114/2012	JX913067
2012	Delaware/04/2012	KC892616
2012	Delhi/9971/2012	KF952362
2012	Ekaterinburg/RII06/2012	JX978770
2012	Florida/02/2012	KC892790
2012	Georgia/05/2012	KC892564
2012	Guangdong/1078/2012	CY125677
2012	Guangdong/1104/2012	CY125679
2012	Guangdong/1154/2012	CY125681
2012	Guangdong/947/2012	CY125691
2012	Hormozgan/807/2012	KC865621
2012	Houston/JMM_67/2012	CY182737
2012	Japan/3060/2012	CY130200
2011	Maine/05/2011	KC882525
2011	Maryland/17/2011	KC882446
2011	Massachusetts/07/2011	KC882516
2011	Mexico/VER60/2011	KJ855341
2011	Nebraska/08/2011	KC882625
2011	Netherlands/063/2011	CY114423
2011	New Jersey/04/2011	KC882520
2011	Oklahoma/02/2011	KC883074
2011	Pennsylvania/12/2011	KC882448
2011	Singapore/EN122/2011	CY124152
2011	Singapore/GP1490/2011	CY124222
2011	Tennessee/F2092c90/2011	CY167319
2011	Victoria/361/2011	KJ942682
2011	Wisconsin/01/2011	KC882810
2011	Zhongshan/01/2011	KJ567660
2011	Zhongshan/02/2011	KJ567661
2012	Alabama/02/2012	KC893091
2012	Alaska/21/2012	KC892659
2012	Alborz/1095/2012	KC865654
2012	Arizona/M14/2012	KJ635922
2012	Boston/DOA2-098/2012	CY148310
2012	California/07/2012	KC893076
2012	Chicago/YGA_04007/2012	CY171073
2012	Colorado/09/2012	KC892466
2012	Czech_Republic/114/2012	JX913069
2012	Delaware/04/2012	KC892617
2012	Delhi/9971/2012	KF952413
2012	Ekaterinburg/RII06/2012	JX978771
2012	Florida/02/2012	KC892789
2012	Georgia/05/2012	KC892565

2012	Kenya/248/2012	KF451875
2012	Mexico/VER58/2012	KJ855355
2012	Moscow/RII05/2012	KC488837
2012	Ontario/027/2012	KF551076
2012	Peru/PER051/2012	CY160688
2012	Quebec/005/2012	KF598738
2012	Saint_Petersburg/RII60/2012	JX978776
2012	Santiago/p20d0/2012	KJ943678
2012	Texas/50/2012	KC892248
2012	Zhongshan/01/2012	KJ567656
2012	Zhongshan/02/2012	KJ567657
2013	Alabama/07/2013	KF789571
2013	Alaska/06/2013	KF789569
2013	Arizona/16/2013	KM064402
2013	Boston/DOA2-146/2013	CY148676
2013	California/34/2013	KM063954
2013	Czech_Republic/216/2013	KJ880910
2013	Delhi/1191/2013	KF952369
2013	Florida/21/2013	KF789968
2013	Guangdong/114/2013	MF150435
2013	Guangdong/403/2013	MF150436
2013	Guangdong/424/2013	MF150437
2013	Guangdong/473/2013	MF150438
2013	Guangdong/519/2013	MF150439
2013	Guangdong/54/2013	MF150434
2013	Guangdong/9/2013	MF150433
2013	Helsinki/716/2013	KF805640
2013	Helsinki/824/2013	KF805664
2013	Hokkaido/30-1-a/2013	AB796432
2013	Houston/JMM_102/2013	CY182993

2012	Guangdong/1078/2012	CY125678
2012	Guangdong/1104/2012	CY125680
2012	Guangdong/1154/2012	CY125682
2012	Guangdong/947/2012	CY125692
2012	Hormozgan/807/2012	KC865622
2012	Houston/JMM_67/2012	CY182739
2012	Kenya/248/2012	KF451888
2012	Mexico/VER58/2012	KJ855357
2012	Moscow/RII05/2012	KC488838
2012	Osaka/11K068/2012	KC134369
2012	Peru/PER051/2012	CY160690
2012	Saint_Petersburg/RII60/2012	JX978777
2012	Santiago/p20d0/2012	KJ943680
2012	Texas/50/2012	KC892237
2012	Zhongshan/01/2012	KJ567662
2012	Zhongshan/02/2012	KJ567663
2013	Alabama/07/2013	KF790150
2013	Arizona/16/2013	KM064399
2013	Boston/DOA2-146/2013	CY148678
2013	California/34/2013	KM064204
2013	Czech_Republic/216/2013	KJ880912
2013	Delhi/1191/2013	KF952418
2013	Florida/21/2013	KF789971
2013	Guangdong/114/2013	MF150442
2013	Guangdong/403/2013	MF150443
2013	Guangdong/424/2013	MF150444
2013	Guangdong/473/2013	MF150445
2013	Guangdong/519/2013	MF150446
2013	Guangdong/54/2013	MF150441
2013	Guangdong/9/2013	MF150440

2013	Indiana/01/2013	KF790345	2013	Helsinki/716/2013	KF805638
2013	Iowa/08/2013	KM064205	2013	Helsinki/824/2013	KF805662
2013	Kenya/254/2013	KF451881	2013	Hokkaido/30-1-a/2013	AB796433
2013	Korea/3419/2013	CY147311	2013	Houston/JMM_102/2013	CY182995
2013	Massachusetts/16/2013	KM064207	2013	Indiana/01/2013	KF789570
2013	New_Jersey/08/2013	KM064469	2013	Iowa/08/2013	KM063965
2013	New_York/32/2013	KM064361	2013	Kenya/254/2013	KF451893
2013	Quebec/8118/2013	KF685747	2013	Massachusetts/16/2013	KM064199
2013	Santiago/p36d0/2013	KJ942943	2013	New_Jersey/08/2013	KM064464
2013	Sao_Paulo/11079/2013	KF142476	2013	New_York/32/2013	KM064216
2013	Singapore/H2013.060/2013	KF432083	2013	Quebec/8118/2013	KF685746
2013	Suzhou/1275/2013	KF034879	2013	Santiago/p36d0/2013	KJ942945
2013	Switzerland/9715293/2013	540526(GISAID)	2013	Singapore/H2013.060/2013	KF432085
2013	Tehran/44476/2013	KJ196075	2013	Suzhou/1275/2013	KF034881
2013	Zhongshan/01/2013	KJ567658	2013	Switzerland/9715293/2013	540525(GISAID)
2013	Zhongshan/02/2013	KJ567659	2013	Tehran/44476/2013	KJ196079
2014	Alaska/26/2014	KM852977	2013	Zhongshan/01/2013	KJ567664
2014	British_Columbia/83/2014	KP701718	2013	Zhongshan/02/2013	KJ567665
2014	British_Columbia/91/2014	KP701724	2014	Alaska/26/2014	KM852978
2014	California/02/2014	KM064121	2014	California/02/2014	KM064137
2014	California/NHRC421194/2014	KJ938667	2014	California/NHRC421194/2014	KJ938668
2014	Colorado/11/2014	KM063986	2014	Colorado/11/2014	KM064524
2014	Czech_Republic/1/2014	KJ561706	2014	Czech_Republic/1/2014	KJ561708
2014	Delaware/07/2014	KM064001	2014	Delaware/07/2014	KM064241
2014	Georgia/02/2014	KM064526	2014	Georgia/02/2014	KM063944
2014	Guangdong/671/2014	MF113163	2014	Guangdong/671/2014	MF113184
2014	Guangdong/704/2014	MF113164	2014	Guangdong/704/2014	MF113185
2014	Guangdong/748/2014	MF113166	2014	Guangdong/748/2014	MF113186
2014	Guangdong/753/2014	MF113165	2014	Guangdong/753/2014	MF113187
2014	Guangdong/802/2014	MF113167	2014	Guangdong/802/2014	MF113188

2014	Hokkaido/M1/2014	AB934036	2014	Hokkaido/M1/2014	AB934037
2014	Hong_Kong/4801/2014	578430(GISAID)	2014	Hong_Kong/4801/2014	578429(GISAID)
2014	Japan/3760/2014	CY187678	2014	Jeonnam/1645/2014	KP144953
2014	Japan/3761/2014	CY187679	2014	Minnesota/02/2014	KM064278
2014	Korea/3773/2014	CY187691	2014	New_York/08/2014	KM063957
2014	Korea/3784/2014	CY187702	2014	Virginia/10/2014	KM063987
2014	Minnesota/02/2014	KM064273	2015	Alabama/09/2015	KT842002
2014	New_York/08/2014	KM064046	2015	Alaska/30/2015	KT843786
2014	Ontario/66/2014	KP701641	2015	Arizona/09/2015	KT844368
2014	Virginia/10/2014	KM063999	2015	Bangkok/SI-MI01/2015	KP877373
2015	Alabama/09/2015	KT842000	2015	Bangkok/SI-MI21/2015	KP877393
2015	Alaska/30/2015	KT843784	2015	Bangkok/SI-MI25/2015	KP877397
2015	Arizona/09/2015	KT844366	2015	Bangkok/SI-MI32/2015	KP877404
2015	Bangkok/SI-MI01/2015	KP877341	2015	California/82/2015	KT843474
2015	Bangkok/SI-MI32/2015	KP877372	2015	Delaware/13/2015	KT842507
2015	British_Columbia/05/2015	KP701737	2015	Florida/52/2015	KT843658
2015	British_Columbia/11/2015	KP701743	2015	Florida/78/2015	KU591776
2015	California/82/2015	KT843424	2015	Guangdong/116/2015	MF113193
2015	Czech_Republic/27/2015	KR534285	2015	Guangdong/138/2015	MF113194
2015	Czech_Republic/38/2015	KR534297	2015	Guangdong/188/2015	MF113195
2015	Delaware/13/2015	KT842393	2015	Guangdong/55/2015	MF113190
2015	Florida/52/2015	KT843651	2015	Guangdong/580/2015	MF113196
2015	Florida/78/2015	KU591774	2015	Guangdong/583/2015	MF113197
2015	Guangdong/116/2015	MF113172	2015	Guangdong/616/2015	MF113198
2015	Guangdong/138/2015	MF113173	2015	Guangdong/716/2015	MF113199
2015	Guangdong/188/2015	MF113174	2015	Guangdong/724/2015	MF113200
2015	Guangdong/55/2015	MF113169	2015	Guangdong/743/2015	MF113201
2015	Guangdong/580/2015	MF113175	2015	Guangdong/747/2015	MF113202
2015	Guangdong/583/2015	MF113176	2015	Guangdong/8/2015	MF113189
2015	Guangdong/616/2015	MF113177	2015	Guangdong/85/2015	MF113191

2015	Guangdong/716/2015	MF113178
2015	Guangdong/724/2015	MF113179
2015	Guangdong/743/2015	MF113180
2015	Guangdong/747/2015	MF113181
2015	Guangdong/8/2015	MF113168
2015	Guangdong/85/2015	MF113170
2015	Guangdong/97/2015	MF113171
2015	Indiana/14/2015	KT843492
2015	Japan/4520/2015	CY193773
2015	Japan/4763/2015	CY194016
2015	Maine/20/2015	KT842854
2015	Massachusetts/18/2015	KT843063
2015	Massachusetts/25/2015	KU590433
2015	New_Jersey/49/2015	KT843367
2015	New_York/20/2015	KT842017
2015	Oregon/16/2015	KT844307
2015	Quebec/38/2015	KU729489
2015	Texas/43/2015	KT842876
2015	Texas/73/2015	KX003463
2015	Washington/61/2015	KX003458
2016	Alabama/09/2016	KX415704
2016	Arizona/37/2016	KX413102
2016	California/21/2016	KX414329
2016	California/98/2016	KX416688
2016	Colorado/27/2016	KX416520
2016	Florida/06/2016	KX414081
2016	Florida/55/2016	KX416864
2016	Georgia/45/2016	KX413294
2016	Guangdong/12/2016	MF113182
2016	Guangdong/264/2016	MF113183
2015	Guangdong/97/2015	MF113192
2015	Indiana/14/2015	KT843374
2015	Maine/20/2015	KT843326
2015	Massachusetts/18/2015	KT843065
2015	Massachusetts/25/2015	KU590435
2015	New_Jersey/49/2015	KT843351
2015	New_York/20/2015	KT842471
2015	Oregon/16/2015	KT844309
2015	Texas/43/2015	KT842914
2015	Texas/73/2015	KX003465
2015	Washington/61/2015	KU591231
2016	Alabama/09/2016	KX415706
2016	Arizona/37/2016	KX413104
2016	California/21/2016	KX414331
2016	California/98/2016	KX416690
2016	Colorado/27/2016	KX416522
2016	Florida/06/2016	KX414083
2016	Florida/55/2016	KX416866
2016	Georgia/45/2016	KX413296
2016	Guangdong/12/2016	MF113203
2016	Guangdong/264/2016	MF113204
2016	Illinois/12/2016	KX416674
2016	Indiana/25/2016	KX415018
2016	Maine/03/2016	KX413072
2016	Massachusetts/28/2016	KX416562
2016	Michigan/70/2016	KX416770
2016	New_Jersey/18/2016	KX416586
2016	New_York/29/2016	KX416282
2016	North_Carolina/44/2016	KX416930
2016	Texas/131/2016	KX416978

2016	Illinois/12/2016	KX416672	2016	Texas/49/2016	KX415530
2016	Indiana/25/2016	KX415016	2016	Virginia/42/2016	KX416266
2016	Maine/03/2016	KX413070	2016	Washington/52/2016	KX416826
2016	Massachusetts/28/2016	KX416560	2016	Wisconsin/62/2016	KX416994
2016	Michigan/70/2016	KX416768	2016	Wyoming/14/2016	KX416498
2016	New_Jersey/18/2016	KX416584			
2016	New_York/29/2016	KX416280			
2016	North_Carolina/44/2016	KX416928			
2016	Texas/131/2016	KX416976			
2016	Texas/49/2016	KX415528			
2016	Virginia/42/2016	KX416264			
2016	Washington/52/2016	KX416824			
2016	Wisconsin/62/2016	KX416992			
2016	Wyoming/14/2016	KX416496			

*Gene sequences of Switzerland/9715293/2013 and Hong Kong/4801/2014 were accessed from GISAID EpiFl database.
